# Gradient Boosted Machine Learning Model to Predict H_2_, CH_4_, and CO_2_ Uptake in Metal–Organic Frameworks Using Experimental Data

**DOI:** 10.1021/acs.jcim.3c00135

**Published:** 2023-07-18

**Authors:** Tom Bailey, Adam Jackson, Razvan-Antonio Berbece, Kejun Wu, Nicole Hondow, Elaine Martin

**Affiliations:** † School of Chemical and Process Engineering, University of Leeds, Leeds LS2 9JT, U.K.; ‡ Zhejiang Provincial Key Laboratory of Advanced Chemical Engineering Manufacture Technology, College of Chemical and Biological Engineering, Zhejiang University, Hangzhou 310027, China

## Abstract

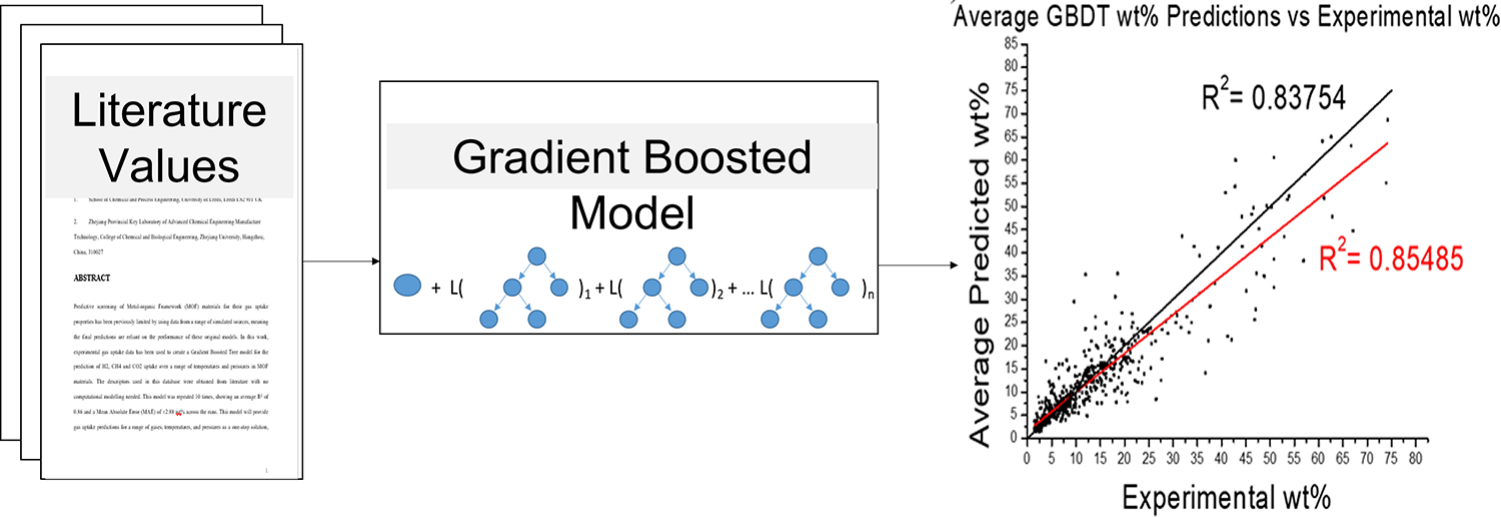

Predictive screening of metal–organic framework (MOF) materials for their gas uptake properties has been previously limited by using data from a range of simulated sources, meaning the final predictions are dependent on the performance of these original models. In this work, experimental gas uptake data has been used to create a Gradient Boosted Tree model for the prediction of H_2_, CH_4_, and CO_2_ uptake over a range of temperatures and pressures in MOF materials. The descriptors used in this database were obtained from the literature, with no computational modeling needed. This model was repeated 10 times, showing an average *R*^2^ of 0.86 and a mean absolute error (MAE) of ±2.88 wt % across the runs. This model will provide gas uptake predictions for a range of gases, temperatures, and pressures as a one-stop solution, with the data provided being based on previous experimental observations in the literature, rather than simulations, which may differ from their real-world results. The objective of this work is to create a machine learning model for the inference of gas uptake in MOFs. The basis of model development is experimental as opposed to simulated data to realize its applications by practitioners. The real-world nature of this research materializes in a focus on the application of algorithms as opposed to the detailed assessment of the algorithms.

## Introduction

Using porous materials in gas storage has become an increasingly important topic, with effective storage and/or release of gases such as H_2_, CH_4_, and CO_2_ being potentially key in climate change mitigation.^1−3^ Porous materials, with large surface areas and open spaces, allow for higher uptakes of gas at lower pressures when compared to using traditional bottles.^4^ Metal–organic framework (MOF) materials have been shown previously to be highly successful in gas absorption^5^ and in particular are more suited to absorption than other porous materials, such as zeolites, due to an absence of dead volume in the structures, which leads to a higher efficiency.^6^ MOF crystalline structures comprise repeating metals containing secondary building units (SBUs) joined together by organic linkers. The SBUs and linkers can potentially be combined in an almost limitless number of ways, allowing for extensive design for the application required.^6^ As a result of this, computational screening for MOF materials becomes important to save time and efficiently find a structure suited to the desired application, such as gas uptake/storage. Previous work by Pardakhti et al. created a random forest (RF) model to predict the methane uptake in ∼130,000s simulated MOF structures,^7^ using descriptors gained through Grand Canonical Monte Carlo (GCMC) modeling, such as void fraction, surface area, and density. This model had a high predictive performance, with a coefficient of determination (*R*^2^) of 0.98 and a mean average percentage error (MAPE) of 7.18. However, this model is limited by only predicting for uptake at 35 bar and 298 K, limiting its use for researchers. More recently, Fanourgakis et al. made an RF-based model to predict CH_4_ and CO_2_ uptake in ∼78,000 structures and achieved an *R*^2^ of 0.96 for predictions on a test set.^8^ A key improvement on the previous work is the ability to predict for two separate gases (CH_4_ and CO_2_) and at a range of pressures (1–65 bar for CH_4_, 0.05–2.5 bar for CO_2_).

RF models are ensembles of decision trees (DTs), with the combination of many DTs improving the model performance and decreasing certain limitations found in DTs. Briefly, DTs are a simple class of machine learning models that start with all of the prediction data being held in a root node, which is then sequentially split through binary decisions by internal nodes until it reaches a terminal node, which will be the prediction.^9^ However, if each output for the training data has a corresponding node, while the performance for the training set is very high, it may struggle to predict new data. To counter this, a “minimum leaf size” can be set, where the value for the terminal node will be the average of several outputs rather than just one, with the number of outputs being averaged corresponding to the “minimum leaf size”. This will result in a lack of performance on the training set but should give a model that is more flexible toward new data.

Ensemble models, such as RFs of gradient boosted decision trees (GBDTs), allow for a more flexible model while avoiding loss of performance. RFs fit many trees (usually hundreds or thousands), with the average prediction from the trees being given.^10^ With the average being taken over many trees, it allows for the individual trees to be weaker, to limit overfitting to the training data, with the average prediction over many trees increasing the performance. GBDT is also a technique that uses many decision trees, but rather than have the trees be separate from each other, the trees are built based on the previous iteration to slowly approach a model with high performance.^9,11^ This is achieved by the model first taking the average of all of the output data and then finding the difference of the output values to this average, with these differences being pseudo-residuals. The model will then form a tree to predict for these residuals and not the actual outputs. From this tree, a prediction would be the average output value plus/minus the predicted residual. However, just from this first tree, there could be predictions that are completely accurate, meaning the model is overfitting to the training data and will have reduced performance with new data. To avoid this, a learning gradient can be applied to the model, which acts as a modifier to the predicted residuals. For example, Predicted Output = Average Output + (Gradient × Predicted Residual). Following this first tree, residuals from these predictions will be used to form the second tree and so on. While this learning gradient does mean that the individual decision trees are much weaker now, by gradually building the model performance, overfitting can be reduced while giving a model with more accurate predictions. Friedman, who developed the gradient boosting model, showed that taking lots of these small steps would lead to a better fitting model while reducing any bias.^12^

These previous models, however, obtained initial gas uptake values and several descriptors using GCMC modeling. This limits the transferability of the data to real-world applications as the gas uptake predictions determined through the machine learning (ML) models may be imperfect due to any errors present in the GCMC models, which, while they might be small, means that the regression model will be starting from a point of error. For researchers looking to predict the gas uptake on a not-yet synthesized MOF, certain physical descriptors, such as pore size and surface area, will only be available through GCMC modeling of the theoretical structure. Since these gas uptake models require these descriptors, researchers would first have to perform these GCMC calculations before a gas uptake prediction could be made.

This work details a predictive ML model for the uptake of multiple gases (H_2_, CH_4_, CO_2_) at a range of temperatures (30–333 K) and pressures (0.06–100 bar). For researchers to use this model for unsynthesized materials, this model will need to be of comparable performance to a previous work while only using predictors that can be gained without the use of GCMC modeling/having already performed a gas isotherm (such as pore size/surface area). The gas uptakes will be obtained from previously published results to remove the errors of GCMC modeling, thus providing an easy-to-use predictive tool for new researchers. The developed ML model shows a high predictive performance while allowing for a range of different predictions to be performed for a single MOF structure. Partial least-squares (PLS) regression was performed to indicate what descriptors are the most significant in the prediction of gas uptake.

## Methods and Materials

A database was formed using experimental gas uptake data from previously published papers, with a full list of MOF materials and their corresponding references provided in the Supplementary Information. The data were collected by manually searching and reading these papers, giving a total of 589 datapoints, with some datapoints being from the same MOF material but with different gases, temperatures, or pressures used. This data was selected from what was available at the time while ensuring that the uptakes were not from papers where the aim was to synthesize defective forms of these MOFs as the model would not be able to account for this currently. The datapoints are split into 205 for H_2_ uptake, 268 for CO_2_ uptake, and 115 for CH_4_ uptake, corresponding to 304 unique MOFs. The aim was to form a database that represented a wide range of MOF structures while giving multiple datapoints to each MOF structure where possible (with variation in the gas absorbed, temperature, and pressure). The wt % values ranged from 1.5 to 74.2 wt %, the temperature ranged from 30 to 313 K, and the pressure ranged from 0.1 to 100 bar. By only using gravimetric uptake data, either through collection or calculation from the literature, and avoiding papers where the MOF produced was purposefully defective, the literature available was limited. This meant that database formation was a time-consuming process and a limiting factor in database size, alongside what literature was available.

Gravimetric uptake data was used rather than volumetric data for ease of comparison. The unit used in this work was weight percentage (wt %) uptake, with some values calculated from cm^3^ g^–1^ using the density of the gas. The wt % was calculated using eq 1:
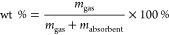
1where *m*_gas_ is the mass of gas absorbed and *m*_absorbent_ is the mass of the absorbent. It was found that different published results for wt % were calculated in two possible ways, with either eq 1 or by simply dividing the absorbed gas by the weight of the absorbent. At low uptakes (such as those for H_2_ absorption), the difference between these two values is small, but at larger uptakes (such as those found for CO_2_ and CH_4_), the difference between the two values is considerable. These values were converted to the same measure, using eq 1, to ensure they are comparable and reduce the data range entering the predictive model, which should lead to easier fitting of the data.^13^

The descriptors used can be divided into three categories: (1) the type and number of bonds present in the linker unit, (2) the metal present in the SBU, and (3) other physical/chemical conditions for the gas absorption (type of gas, temperature, pressure, electronegativity difference between the MOF and the gas). Textural features, such as surface area and pore size, were purposefully not included here to ensure future users would not need to perform other computational modeling before using this model. Overall, 51 descriptor variables (Table 1) were used, with the output being the natural log of the gas uptake wt %. This natural log was used to account for unequal spacing between datapoints.

**Table 1:** List of Descriptors Used in Machine Learning Models

type of descriptor	list of descriptors
primary building units (PBUs)	C–C, C–C (ring), (ring) C–C (ring), C=C, C–O, C=O, C–N, C=N (ring), N–N (ring), N=N (ring), N=N (ring), (ring) C–O, (ring) C=O, (ring) C–S (ring), (ring) N–S (ring), (ring) C–N, C–N (ring), (ring) C=C (ring), (ring) N–C (ring), (ring) N=C (ring), C≡C, C≡N, N–O, N=O, O–R, C–R, (ring) C–R
secondary building units (SBUs)	Al, Cd, Co, Cu, Mg, Mn, Ni, Zr, Zr_4_O, Sc, Ti, Be, Pd, Y, Er, In, Cr, Fe, Mo, Zn
physical conditions (PHYS)	largest electronegativity difference, temperature (k), pressure (bar), gas molecular weight (g/mol)

Several machine learning methods, linear regression, quadratic support vector machine (SVM), DT, and gradient boosted decision trees (GBDTs), were fitted and tested. In lieu of using an external test set, 10-fold cross-validation was used, with the low amount of data available making it impossible to choose a test set without bias. Machine learning research, performed in relation to materials engineering, has utilized cross-validation as opposed to an external test set for validation due to a relative lack of data available.^14−17^ The GBDT model had several hyperparameters (number of trees, minimum leaf size, and learning rate) manually optimized to give the lowest mean squared error (MSE) on each fold when used as a validation set. This optimization led to a GBDT model with 600 trees, a learning rate of 0.05, and a minimum leaf size of 3. During optimization, increasing the minimum leaf size from 1 to 2 to 3 did not improve the *R*^2^ significantly as anticipated, with the value decreasing marginally as the leaf size was increased (0.8709 to 0.8669 to 0.8643). However, while it has the lowest *R*^2^ value, a leaf size of 3 was utilized to ensure that if new data is included in the future, this added flexibility should reduce potential overfitting. The linear regression, DT, and quadratic SVM models had their hyperparameters optimized using the “OptimiseHyperparameters″ function in MATLAB 2020. The full list of hyperparameters is provided in the Supporting Information.

Each model was run 10 times to give a varied split of the different folds, ensuring that each model was repeatable even when the folds changed. These models were then evaluated by their average *R*^2^ values, the average validation fold MSE (KFold Loss), and the average mean absolute error (MAE) for when the predicted data was converted back from being a logged value and compared with the original value. This MAE was done for each gas as well, to give a more accurate scale of error. Alongside the MAE, the mean absolute percentage error (MAPE) was also calculated to give a relative measure of error.

## Results and Discussion

**Figure 1:**
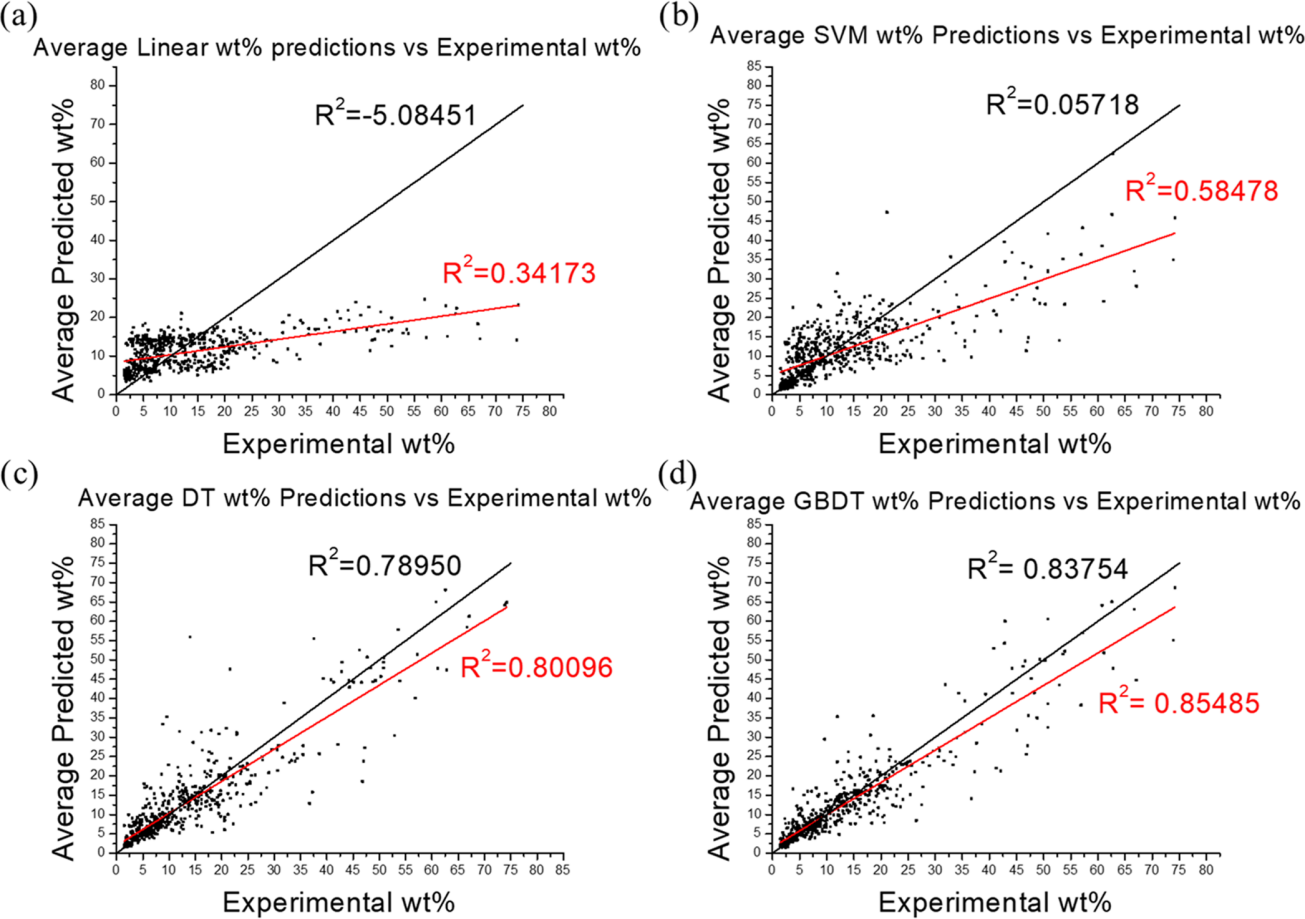
Regression plots for the developed ML models: (a) linear model; (b) SVM; (c) DT model; and (d) GBDT model. Each plot uses the average prediction for each datapoint (over 10 runs) versus the real experimental wt % values found in the literature. The black line is *y* = *x*, with the *R*^2^ around this line calculated and shown in black text. The red line is a fitted line of the best fit, with the *R*^2^ for this shown in red text.

The average *R*^2^, KFold Loss, MAE, and MAPE from the four ML methods while predicting for all gases are listed in Table 2, with the regression plots for each model shown in Figure 1. The regression plots were made by converting the prediction and target wt % values back from natural logs and then taking the average of the prediction values for each datapoint over 10 runs.

**Table 2:** *R*^2^, Validation MSE (KFold Loss), MAE, and MAPE for Each of the Machine Learning Models Used^a^

method	average *R*^2^	average KFold loss	average MAE	average MAPE
linear	0.330	0.605	7.251	87.822
SVM	0.650	0.305	5.309	51.381
DT	0.777	0.195	3.790	35.853
GBDT	0.864	0.117	2.882	26.544

a MAE and MAPE were calculated once the data was converted back from a log value.

The GBDT model shows the highest level of performance across the board (*R*^2^ = 0.86, average KFold loss = 0.117, average MAE = 2.882 wt %, average MAPE = 26.54%), which is to be expected from a more complex machine learning model. The KFold loss being the lowest shows this model to be the best at predicting new data, with the lowest MSE for the held-out folds, which is key for a new researcher to use this model. In relation to previous literature examples by Pardakhti et al. and Fanourgakis et al.,^7,8^ this does show a slightly lower level of performance (*R*^2^ = 0.86 compared to 0.98 or 0.96 respectively), but with the added flexibility available for this model in which multiple gases and conditions can be predicted, making it a success. The GBDT performed consistently across the 10 runs, with the relative standard deviation for each error shown in Table 3.

**Table 3:** Relative Standard Deviation (%) for *R*^2^, KFold Loss, MAE, and MAPE across the 10 Runs

	*R*^2^	KFold loss	MAE	MAPE
relative standard deviation (%)	0.6	3.6	1.4	1.9

In terms of MAPE, there is a deviation from the model by Pardakhti et al., with 26.544% compared to 7.18%. Again, however, with the limited data used and the flexibility of the model formed for a new user, it is still a success. The predictions for this work being based on previous literature results should also give predictions that are more applicable in a real-world setting. An average MAE of ±2.882 wt % is given for all of the datapoints, but there is variation depending on the gas being predicted (Table 4), which new researchers can apply to their predictions. Note here that these errors are for the specific datapoints for different gases when predictions are being performed on the full data set, not for separate models for each gas. Being able to perform calculations for any of the gases while not changing the training database is a key aspect of the model’s flexibility.

**Table 4:** Average MAE and Average MAPE when Fitting Data for Each Gas in the GBDT Model, over 10 Runs

gas type	average MAE	average MAPE
H_2_	0.759	20.70%
CO_2_	4.598	32.26%
CH_4_	2.667	23.64%
all gases	2.882	26.54%

GBDT is the most accurate model, and fitting was repeated while limiting the descriptors used to examine how each category contributed to the fitting. For each of these, the adjusted *R*^2^ was also collected to observe if overfitting through the number of descriptors was occurring (Table 5). Adjusted *R*^2^ is calculated using eq 2 and is used to measure *R*^2^ in relation to the number of descriptors used, only increasing if the increase in *R*^2^ is significant in relation to the increase in descriptors.^18^
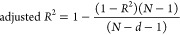
2

**Table 5:** Comparison of *R*^2^, Adjusted *R*^2^, Kfold Loss, Average MAE, and Average MAPE when Different Combinations of Primary Building Unit (PBU), Secondary Building Unit (SBU), and Physical Conditions (PHYS) Were Used in the Fitting of the GBDT Model^a^

	average *R*^2^	average adjusted *R*^2^	average Kfold loss	average MAE	average MAPE
PBU (27)	0.062	0.017	0.9016	8.200	100.600
SBU (20)	0.023	–0.012	0.848	8.280	97.180
PHYS (4)	0.743	0.741	0.222	4.431	40.858
PHYS + PBU (31)	0.842	0.8340	0.1356	3.217	29.049
PHYS + SBU (24)	0.803	0.795	0.1702	3.646	34.001
PBU + SBU (47)	0.064	–0.017	0.9100	8.324	102.249
**all descriptors (51)**	0.864	0.851	0.117	2.882	26.544

a The set of descriptors with the highest adjusted *R*^2^ has been highlighted. The number of descriptors used in each category is shown in brackets.

As can be seen in Table 5, the physical uptake conditions (PHYS; pressure, temperature, type of gas, electronegativity difference) play the biggest role in the prediction for the overall uptake, which is understandable as the way a gas behaves is affected drastically by the environment, as seen in the ideal gas equation for example. Following this, predicting using just the primary building unit (PBU) descriptors gives the next most accurate predictions (when using one category of predictors at a time), with the SBU descriptors being the least accurate. When combining these descriptors, the model with the highest predictive performance is formed, with the highest adjusted *R*^2^, indicating that overfitting through too many descriptors is not occurring. If this database is expanded, leading to an increase in runtimes, then limiting to the physical conditions and the PBU descriptors, which would reduce the predictors from 51 to 31, could give a comparatively accurate result in less time.

**Figure 2:**
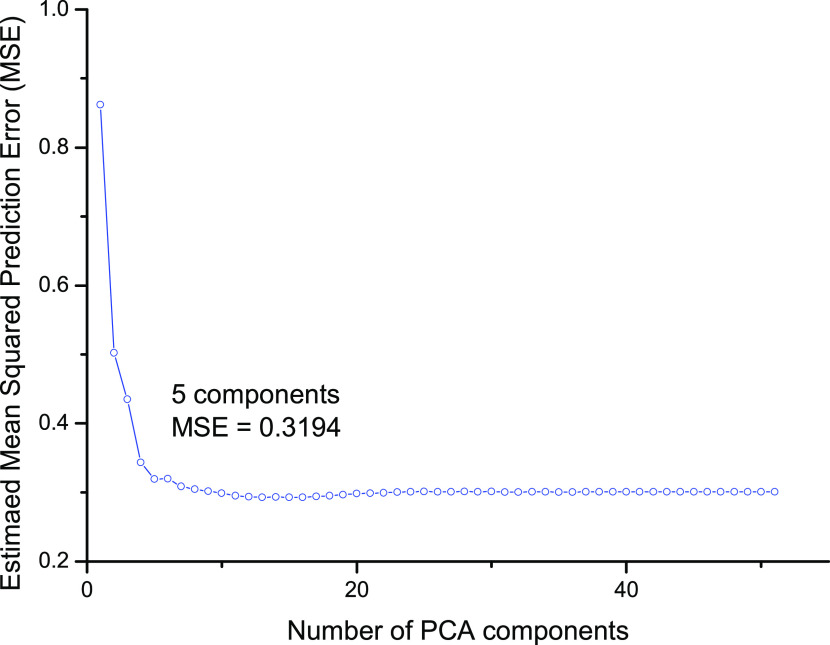
Estimated mean squared prediction error vs number of PLS components. The datapoint at five components has been highlighted.

Partial least-squares (PLS) fitting was performed to give variable importance scores (VIPScores) for each descriptor, with a higher score meaning that the descriptor contributes more to the percentage variance explained. First, PLS was performed to find the minimum number of components needed for the model to predict accurately. The results for this are shown in Figure 2, with the estimated mean squared prediction error plotted against the number of components used.

**Figure 3:**
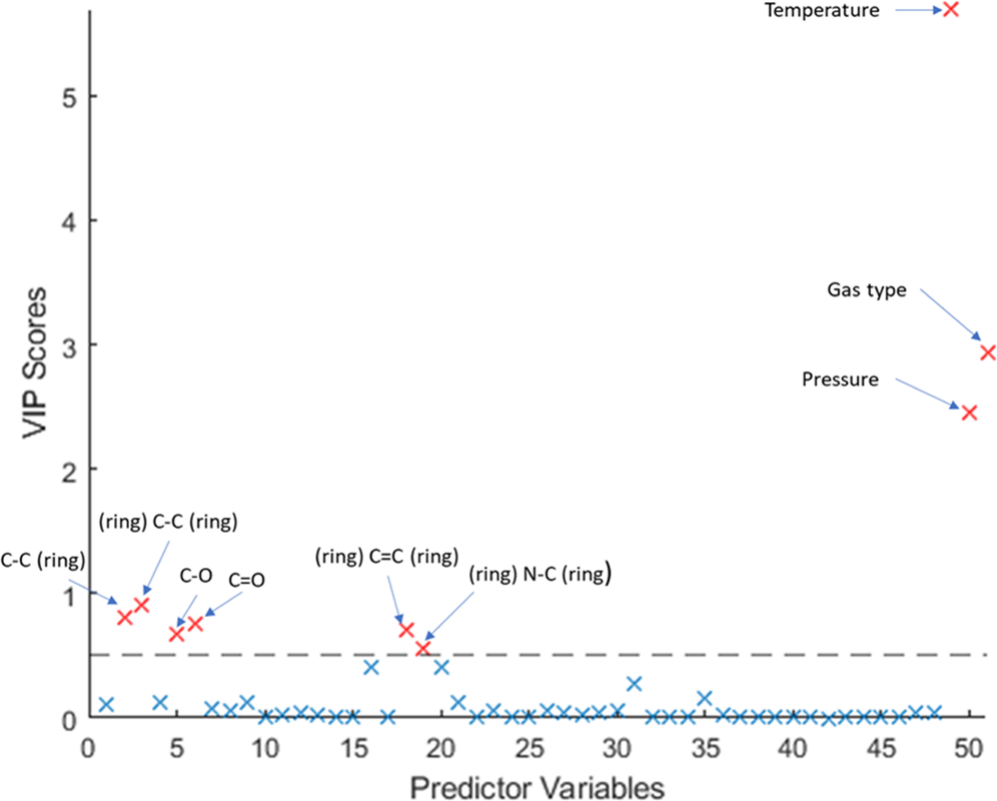
Variable importance scores for each of the 51 descriptors. Those with a score > 0.5 are labeled and highlighted in red.

In this principle component analysis (PCA) plot (Figure 2), the “elbow”, being the point at which the error starts to level off, is at seven components, with the elbow method of choosing the number of components being well documented.^19^ This method is performed to ensure that overfitting is not occurring through including too many components and because after this point the increase in performance for increasing components has been reduced drastically. Following this, PLS was repeated using six components to give accurate VIPScores for the descriptors, which should be comparable to the variable importance found earlier when using different data sets. These VIPScores are shown in Figure 3, with the descriptors showing a score of 0.5 or higher labeled. While in the literature a score > 1 is used as an indication that a descriptor is important,^20^ this would only leave the temperature, pressure, and type of gas in this case. As shown in Table 4, using other descriptors alongside the physical conditions does increase the performance of the model while not overfitting, as seen with the increasing adjusted *R*^2^, so some of these must also be important.

After the physical conditions (except electronegativity difference), the descriptors that show the highest contribution are those relating to certain linker bonds in the PBUs as highlighted in Figure 3. The bonds with the highest contribution to the uptake being carbon-bonded to other atoms make sense as a higher number of C–C bonds for example would usually result in a longer linker, increasing the surface area and the pore size.^21^ Fitting the GBDT model was repeated using these descriptors with VIPScores > 1 and > 0.5 to see how their inclusion affected performance, with their errors shown in Table 6.

**Table 6:** *R*^2^, Adjusted *R*^2^, Kfold Loss, MAE, and MAPE for GBDT Models Fitting Using only the Descriptors with VIPScores > 1, VIPScores > 0.5, and Fitting Using All 51 Descriptors

	*R*^2^	adjusted *R*^2^	Kfold loss	average MAE	average MAPE
VIPScores > 1	0.737	0.736	0.228	4.521	41.415
VIPScores > 0.5	0.804	0.801	0.169	3.651	33.139
all descriptors	0.864	0.851	0.117	2.882	26.544

When limited to these nine descriptors, the model has comparable performance to that found when using the full 51, whereas only using the physical conditions yield a model with a lower performance. Future work using larger databases could benefit from using just these nine descriptors to reduce the computing power required.^13^

An interesting finding from the PLS and fitting the GBDT model with certain descriptor sets is that the SBU metal type shows a very low impact on the predicted gas uptake. This is unexpected as the metal type is one of the key features of an MOF structure so was thought necessary to include in prediction of the gas uptake. There are two potential reasons for this lack of impact. First, the type of metal is not as important as the linker bonds that are present when it comes to gas uptake, with longer/larger linker units potentially leading to higher surface areas/pore sizes. In general, higher surface areas and/or pore volumes will lead to higher gas uptakes so this does make sense why they are so important. The second reason could be due to the limited data set that is present in this work, with a larger data set potentially showing trends for the metal type that cannot currently be seen for this model.

With the completed GBDT model formed, new researchers can use this database and model to form gas uptake predictions on new MOF structures quickly and easily as a one-stop preliminary model. This model differs from others through its flexibility, being able to predict for different gases, temperatures, and pressures without the researcher first needing to perform any other modeling work, only needing to provide the descriptors for the linker, SBU, and the physical conditions for the gas uptake. The use of experimental data in the model fitting should provide results that are more in line with real-world observations, rather than theoretical structures. The errors found for each gas have been provided so researchers using this model may accurately determine a predicted uptake range for their chosen MOF and gas. Future work expanding this database, especially with datapoints at the temperature/pressure extremes, will help improve the performance of this model as it is a relatively small data set compared to other works.^7,8^

## Conclusions

In this work, a GBDT model has been developed to predict the uptake of H_2_, CO_2_, and CH_4_ in MOF materials and is able to predict these for a range of temperatures and pressures. The average *R*^2^ of this model is found to be 0.864 with an average MAE of ± 2.88 wt % for the uptakes. This model’s high performance while using experimental data should provide researchers with predictions more in line with real-world observations, with the added flexibility to vary physical parameters quickly and easily. Future work should aim to expand this database to give greater predictive performance.

## Data and Software Availability

A list of all of the literature data used in this work is provided in a PDF file, which lists the MOF, the physical conditions for the uptake value (temperature and pressure), the wt % value, and the reference for this datapoint. Also, in this document are the hyperparameters used in other machine learning techniques, individual coefficient of determination values for each fold in 10 runs of the GBDT model, and a full list of the references used for the uptake values. An Excel file has been made available with all of the descriptors for each datapoint attached as well, which was used to perform the fitting of the GBDT model. Alongside this, the code used to perform this work has been made available in a ZIP file attached, with annotation provided throughout to explain certain parts. The software needed to perform the model fitting was MATLAB 2020 with the Statistics and Machine Learning toolbox.

## Supporting Information Available

Supporting information document, containing the hyperparameters used in other machine learning techniques, uptake values, and references for all of the data used in fitting the model and the individual coefficient of determination values for each fold in 10 runs of the GBDT model (PDF)
Excel file containing all of the descriptor values for each datapoint used in fitting the GBDT model (XLSX)
Zip file containing code used for this work, with annotation provided throughout (ZIP)

## Abbreviations

DT: decision tree
GBDT: gradient boosted decision tree
GCMC: grand canonical monte carlo
MAE: mean absolute error
MAPE: mean absolute percentage error
ML: machine learning
MOF: metal–organic framework
MSE: mean squared error
PBU: primary building unit
PCA: principal component analysis
PHYS: physical conditions
PLS: partial least squares
SBU: secondary building unit
SVM: support vector machine
VIPScores: variable importance scores
