# Supplementary material for: Gradient Boosted Machine Learning Model to Predict H2, CH4, and CO2 Uptake in Metal–Organic Frameworks Using Experimental Data
Source: J Chem Inf Model. 2023 Jul 18;63(15):4545–51. doi: 10.1021/acs.jcim.3c00135 (PMC10428209; doi:10.1021/acs.jcim.3c00135)
Supplement: Supplementary file 1 — ci3c00135_si_001.pdf [file ci3c00135_si_001.pdf]

Supporting Information

Gradient boosted machine learning model to predict H_2, CH_4 and CO_2 uptake in metal organic frameworks using experimental data

Tom Bailey,[1] Adam Jackson,[1] Razvan-Antonio Berbece,[1] Kejun Wu,*[1,2] Nicole Hondow,*[1] and Elaine Martin*[1]

1. School of Chemical and Process Engineering, University of Leeds, Leeds LS2 9JT UK

2. Zhejiang Provincial Key Laboratory of Advanced Chemical Engineering Manufacture Technology, College of Chemical and Biological Engineering, Zhejiang University, Hangzhou, China, 310027

Email Addresses for Corresponding Authors:

Kejun Wu: k.j.wu@leeds.ac.uk

Nicole Hondow: n.hondow@leeds.ac.uk

Elaine Martin: e.martin@leeds.ac.uk

Table S1: MOF structures with literature H_2 uptakes

MOF Name	Common Name	Pressure (Bar)	Temperature (K)	Uptake (%wt)	Reference
$Be_{12}(OH)_{12}(BTB)_4$		1	77	1.6	Sumida et al., 2009[1]
		20	77	6	
		100	77	9.2	
		95	298	2.3	
Cd4(TCPM)2		1	77	2.8	Chun et al., 2004[2]
Co(BDP)		30	77	3.1	Jin Choi, Dincă and Long, 2008[3]
Co(BTC)(4,4'-bpy)		72	77	2.05	Li.Y et al., 2008[4]
Co2(BDC)2(dabco)		44.2	77	4.11	Suh et al, 2012[5]
Co3(bpdc)3(4,4'-bpy)		1	77	1.98	Lee et al., 2005[6]
Co3(NDC)3(dabco)		1	77	2.45	Chun et al., 2008[7]
Co3[(Co4Cl)3(BTT)8(H2O)12]2		1	77	1.8	Liao et al., 2013[8]
Cr3(BTC)2		1	77	1.9	Sumida, Her, et al., 2011[9]
Cu(dccptp)(NO3)		20	77	1.91	Yang et al., 2008[10]
Cu(peip)		1	77	2.51	Liu, Oh and Soo Lah, 2011[11]
		40	77	4.14	
Cu(pmip)		1	77	2.36	Liu, Oh and Soo Lah, 2011[11]
Cu(TZI)3		1	77	2.4	Nouar et al., 2008[12]
Cu2(BDC)2(dabco)		1	77	1.8	Lee et al., 2007[13]
		33.7	77	2.7	
Cu2(BDDC)		0.95	77	1.64	Bing Zheng et al., 2010[14]
		17	77	3.98	
Cu2(dhtp)	Cu-MOF-74	5	77	2.22	García-Holley et al., 2018[15]
		100	77	3.15	
		100	160	2.87	
Cu2(DAIA)(H2O)2	Cu-MOPF	1	77	2.8	Maity, Karan and Biradha, 2018[16]
Al(OH)(SDC)	CYCU-3-Al	5	77	3.37	García-Holley et al., 2018[15]

		100	77	8.24	
		100	160	7.92	
Fe3(OH)(pbpc)2		1	77	1.6	Jia et al., 2007[17]
		20	77	3.05	
Fe4O2(BTB)8/3		1	77	2.1	Choi et al., 2007[18]
Zn6(BTB)4(4,4’-bpy)3	FJI-1	37	77	6.52	Han et al., 2011[19]
HCu[(Cu4Cl)3(BTT)8]	Cu-BTT	1.2	77	2.42	Dincă et al., 2007[20]
		20	77	4.1	
Cu3(BTC)2	HKUST-1	5	77	3.53	García-Holley et al., 2018[15]
		100	77	5.31	
		100	160	4.91	
Zn4O(CH3PhTDC)3	IFMC-29	1	77	1.75	Cheng et al., 2018[21]
Zn4O(dobdc)2	IRMOF-6	45	77	4.63	Wong-Foy, Matzger and Yaghi, 2006[22]
Zn4O(NDC)3	IRMOF-8	1	77	1.5	Rowsell et al., 2004[23]
Zn4O(HPDC)2	IRMOF-11	1	77	1.62	Rowsell et al., 2004[23]
		33.7	77	3.4	Wong-Foy, Matzger and Yaghi, 2006[22]
Zn4O(pyrdc)2	IRMOF-13	1	77	1.73	Rowsell and Yaghi, 2006[24]
Zn4O(ttdc)2	IRMOF-20	77.6	77	6.25	Wong-Foy, Matzger and Yaghi, 2006[22]
Cd3(bpdc)3	JUC-48	40	77	2.8	Fang et al., 2007[25]
Cu3(BTAT)	MFM-132a	1	77	2.83	Yan et al., 2018[26]
		20	77	6.3	
Mg2(dobdc)	Mg-MOF-74	1	77	2.2	Sumida, Brown, et al., 2011[27]
		100	77	4.9	
Al(OH)(bdc)	MIL-53(Al)	16	77	3.8	Férey et al., 2003[28]
Cr(OH)(bdc)	MIL-53(Cr)	16	77	3.1	Férey et al., 2003[28]
Cr3OF(BTC)2	MIL-100	26.5	77	3.28	Latroche et al., 2006[29]
Cr3O(BDC)2	MIL-101(Cr)	1	77	1.92	Ren et al., 2014[30]
Mn3[(Mn4Cl)3(BTT)8]2	Mn(BTT)	1.2	77	2.2	Dincă et al., 2007[20]
		90	77	6.9	
Zn4O(bdc)3	MOF-5, IRMOF-1	50	77	4.7	Panella et al., 2006[31]

Pd(bdc)	MOF-5 (Pd)	1	77	1.86	Sabo et al., 2007[32]
Zn2(dhtp)	Zn-MOF-74, CPO-27-Zn	1	77	1.77	Rowsell and Yaghi, 2006[24]
		30	77	2.8	Liu et al., 2008[33]
		70	77	7.5	Wong-Foy, Matzger and Yaghi, 2006[22]
Zn4O(BBC)2(H2O)3	MOF-200	80	77	7.4	Furukawa et al., 2010[34]
Zn4O(BTB)4/3(NDC)	MOF-205	80	77	7	Furukawa et al., 2010[34]
Zn4O(BTE)4/3(bpdc)	MOF-210	80	77	8.6	Furukawa et al., 2010[34]
Cu2(bptc)	MOF-505, NOTT-100	1	77	2.47	Chen et al., 2005[35]
		20	77	4.02	Lin et al., 2009[36]
Zn4O(AZD)3	MOF-646	1	77	1.75	Barman et al., 2010[37]
	MOF-808	1	77	2.78	Xia et al, 2016[38]
Ni(dhtp)2	Ni-MOF-74	70	77	1.8	Dietzel et al., 2006[39]
Ni(BTC)(4,4'-bpy)		72	77	3.42	Y. Li et al., 2008[4]
Ni3(OH)(pbpc)3		1	77	1.99	Jia et al., 2007[17]
		20	77	4.15	
Ni3[(Co4Cl)3(BTT)8(H2O)12]2		1	77	1.5	Liao et al., 2013[8]
NJU-bai12-ac		1	77	1.91	Zheng et al., 2013[40]
		20	77	5.9	
Cu2(tptc)	NOTT-101	1	77	2.52	Lin et al, 2009[36]
		20	77	6.06	
		60	77	6.6	
Cu2(qptc)	NOTT-102	1	77	2.24	Lin et al, 2009[36]
		20	77	6.07	
		60	77	7.2	
Cu2(NddIP)	NOTT-103	1	77	2.63	Lin et al, 2009[36]
		20	77	6.51	
		60	77	7.78	
Cu2(DFTP)	NOTT-105	1	77	2.52	Lin et al, 2009[36]

		20	77	5.4	
Cu2(DMTP)	NOTT-106	1	77	2.29	Lin et al, 2009[36]
		20	77	4.5	
Cu2(TMTP)	NOTT-107	1	77	2.26	Lin et al, 2009[36]
		20	77	4.46	
Cu2(ndip)	NOTT-109	1	77	2.33	Lin et al, 2009[36]
		20	77	4.15	
Cu2(pdip)	NOTT-110	1	77	2.64	Yang et al., 2009[41]
		20	77	6.59	
		55	77	7.62	
Cu2(dpdip)	NOTT-111	1	77	2.56	Yang et al., 2009[41]
		20	77	6.48	
		48	77	7.36	
Cu3(BDDC)	NOTT-112	1	78	2.3	Yan et al., 2009[42]
		37.5	77	7.07	
		100	77	8.74	García-Holley et al., 2018[15]
		100	160	8.31	
Cu3(tbtt)	NOTT-113	1	78	2.39	Yan, Blake, et al., 2011[43]
		30	78	5.1	
Cu3(abtt)	NOTT-114	1	78	2.39	Yan, Blake, et al., 2011[43]
		30	78	5	
Cu3(NTBD)	NOTT-115	1	78	2.39	Yan, Blake, et al., 2011[43]
		33	78	5.6	
Cu3(btti)	NOTT-119, PCN-69	1	77	1.44	Yan, Yang, et al., 2011[44]
		44	77	5.6	
Cu4(TDTM)	NOTT-140	1	77	2.5	Tan.C et al., 2011[45]
		20	77	6	
Sc2(bptc)(OH)2	NOTT-400	1	77	2.14	Ibarra et al., 2011[46]
		20	77	3.84	
Sc(TDA)(OH)	NOTT-401	1	77	2.31	Ibarra et al., 2011[46]

		20	77	4.44	
Cu3(ttei)	NU-100	1	77	1.82	Farha et al., 2010[47]
		56	77	9.95	
Cu3(TIPTB)	NU-125, NOTT-122	1	77	2.61	Yan et al., 2014[48]
		5	77	4.6	García-Holley et al., 2018[15]
		100	77	8.2	
		100	160	7.76	
Zr(TBAPy)2	NU-1000	5	77	3.37	García-Holley et al., 2018[15]
		100	77	7.98	
		100	160	7.62	
Zr(Py-XP)	NU-1101	100	77	9.5	Gómez-Gualdrón et al., 2017[49]
Zr(Por-PP)	NU-1102	100	77	9.9	Gómez-Gualdrón et al., 2017[49]
Zr(Py-PTP)	NU-1103	100	77	13	Gómez-Gualdrón et al., 2017[49]
Cu3(TATB)2 (catenated)	PCN-6	1	77	1.9	Sun, Ma, et al., 2006[50]
		50	77	7.2	S. Ma et al., 2008[51]
Cu2(aobtc)	PCN-10	1	77	2.34	X.-S. Wang, Ma, Rauch, et al., 2008[52]
		3.5	30	6.84	
		45	77	5.23	
Cu2(sbtc)	PCN-11	1	77	2.55	X.-S. Wang, Ma, Rauch, et al., 2008[49]
		3.5	30	7.89	
		45	77	5.97	
Cu2(mdip)	PCN-12	1	77	3.05	X.-S. Wang, Ma, Forster, et al., 2008[49]
Cu2(adip)	PCN-14	1	77	2.7	Ma et al., 2009[53]
		45	77	4.42	
Cu2(ebdc)	PCN-16	1	77	2.6	Sun et al., 2010[54]
		45	77	5.1	
Cu2(PMTB)	PCN-21	1	77	1.6	Zhuang et al., 2010[55]
Cu2(bdi)	PCN-46	1	77	1.95	Zhao et al., 2010[56]
		32	77	5.31	

Cu3(btei)	PCN-61	1	77	2.25	Yuan et al., 2010[57]
		33	77	6.24	
Cu3(ntei)	PCN-66	1	77	1.79	Yuan et al., 2010[57]
		45	77	6.65	
Cu3(ptei)	PCN-68, NOTT-116	1	77	1.87	Yuan et al., 2010[57]
		50	77	7.32	
Fe2(abtc)	PCN-250	5	77	3.77	García-Holley et al., 2018[15]
		100	77	5.37	
		100	160	4.93	
Cu2(bdpb)	PMOF-3	1	77	2.47	Zhang et al., 2011[58]
Cu3(TDPAT)	rht-MOF-7	5	77	3.23	García-Holley et al., 2018[15]
		100	77	4.89	
		100	160	4.5	
Sc(BDC)3		1	77	1.5	Perles et al., 2005[59]
Zn4O(NTB)2	SNU-1	1	77	1.9	Young Lee, Yeon Jang and Paik Suh., 2005[60]
Zn2(abtc)(DMF)2	SNU-4	1	77	2.07	Lee et al., 2008[61]
		50	77	3.7	
Cu2(abtc)(DMF)2	SNU-5’	1	77	1.83	Lee et al., 2008[61]
		50	77	5.22	
Cu2(BPnDC)2(4,4’-bpy)	SNU-6	1	77	1.68	Park and Suh., 2008[62]
		70	77	4.87	
Cu2(TCM)	SNU-21S	1	77	1.95	Kim and Suh., 2011[63]
		70	77	4.37	
Zn2(TCPBDA)(H2O)2	SNU-30	61	77	3.27	Park, Cheon and Suh, 2010[62]
Cu2(bdcppi)	SNU-50’	1	77	2.1	Prasad, Hong and Suh, 2010[64]
		1	87	1.39	
		60	77	5.53	
Zn4O(CVB)3	SNU-70	20	77	7	Ahmed et al., 2019[65]
		100	77	10.5	

Zn4O(TCBPA)2	SNU-77H	1	77	1.79	Park H. J. et al., 2011[66]
		90	77	8.1	
Mo3(BTC)2	TUDMOF-1	1	77	1.75	Kramer, Schwarz and Kaskel, 2006[67]
Zr(BPDC)2	UiO-67	5	77	3.23	García-Holley et al., 2018[15]
		100	77	5.86	
		100	160	5.53	
Zr(ACDB)2	UiO-68-Ant	5	77	3.81	García-Holley et al., 2018[15]
		100	77	7.58	
		100	160	7.18	
Zn4O(T2DC)(BTB)4/3	UMCM-2	46	77	6.9	Koh, Wong-Foy and Matzger, 2009[68]
Zn4O(bpdc)1.5(NDC)1.5	UMCM-9	20	77	7.5	Ahmed et al., 2019[65]
		100	77	11.5	
Cu3(BHB)	UTSA-20	1	77	2.9	Guo et al., 2011[69]
		15	77	4.1	
Cu3(bhtc)2	UMCM-150	1	77	2.1	Wong-Foy, Lebel and Matzger, 2007[70]
		45	77	5.7	
Y(BTC)		1	77	1.57	Luo et al., 2008[71]
		10	77	2.1	
Zn(MeIM)2	ZIF-8	55	77	3.01	Zhou et al., 2007[72]
		30	77	3.3	
Co(MeIM)2	ZIF-67	1	77	1.53	Panchariya et al., 2018[73]
Zn(NDC)(bpe)0.5		40	77	2	Chen, Ma, et al., 2006[74]
Zn(peip)		1	77	2.27	Liu, Oh and Soo Lah, 2011b[75]
Zn2(BDC)(tmbdc)(dabco)		1	77	2.08	Chun et al., 2005[76]
Zn2(BDC)2(dabco)		1	77	2.1	Lee et al., 2007[13]
		83.2	77	3.17	Takei et al., 2008[77]
Zn2(btatb)		1	77	2.2	Farha et al., 2008[78]
Zn2(NDC)2(dabco)		1	77	1.7	Chun et al., 2005[76]
Zn2(tftpa)2(dabco)		1	77	1.78	Chun et al., 2005[76]
Zn2(tmbdc)2(4,4'-bpy)		1	77	1.68	Chun et al., 2005[76]

Zn2(tmbdc)2(dabco)		1	77	1.85	Chun et al., 2005[76]
Zn3(bpdc)3(4,4'-bpy)		1	77	1.74	Lee et al., 2005[79]
Zn(5-AT)2	ZTF-1	1	77	1.6	Panda et al., 2011[80]
Zn(Mlai)	IMOF-3	1	77	1.5	Debatin et al., 2010[81]

Table S2: MOF structures with literature CH_4 uptakes

MOF Name	Common Name	Pressure (Bar)	Temperature (K)	Uptake (%wt)	Reference
Al3(TCPT)6	Alsoc-MOF-1	65	270	33.8	Alezi et al., 2015[82]
		65	270	29	
Cd(bpydb)		35	298	5.5	Sharma et al., 2011[83]
Co2(4,4'-bpy)3(NO3)4		30	298	3.56	Kondo et al., 1997[84]
Co2(azpy)3(NO3)4		36	298	2.9	Kondo et al., 2000[85]
Co2(BDC)2(dabco)		35	303	11.4	H. Wang et al., 2008[86]
		75	303	12.28	H. Wang et al., 2008[86]
Co2(dobdc)	Co-MOF-74	35	298	11	Mason, Veenstra and Long, 2014[87]
Cu2(BDC)2(dabco)		35	293	10.8	Seki and Mori, 2002[88]
		35	298	13.2	Seki, 2001[89]
Cu3(BTC)2		35	303	12.7	Senkovska and Kaskel, 2008[90]
Cu3(TDPAT)	Cu-TDPAT	35	298	13.2	Li et al., 2012[91]
Al(OH)(NDC)	DUT-4	35	303	8.8	Senkovska et al., 2009[92]
Al(OH)(bpdc)	DUT-5	35	303	9.8	Senkovska et al., 2009[92]
Co2(NDC)2(dabco)	DUT-8(Co)	60	298	4.5	Klein et al., 2012[93]
Cu2(NDC)2(dabco)	DUT-8(Cu)	60	298	12.8	Klein et al., 2012[93]
Zn2(NDC)2(dabco)	DUT-8(Zn)	60	298	3.7	Klein et al., 2012[93]
Ni5O2(BTB)2	DUT-9	30	298	12	Gedrich et al., 2010[94]
		70	298	17.3	
Zn4O(TCPBDA)3/2	DUT-13	50	298	13.9	Grünker et al., 2011[95]
Co2(bipy)3(BTB)4	DUT-23(Co)	100	298	21.1	Klein et al., 2011[96]
Cu2(BBCDC)	DUT-49	35	298	19.2	Stoeck et al., 2012[97]
Zn6(BTB)4(4,4′-bpy)3	FJI-1	35	273	20.6	Han et al., 2011[19]
		35	298	15.2	
Cu3(BTC)2	HKUST-1	35	298	15.5	Peng, Krungleviciute, et al., 2013[98]
		65	298	17.8	
Zn4O(NH2-bdc)3	IRMOF-3	36.5	298	13	Eddaoudi et al., 2002[99]
Zn4O(C2H4-bdc)3	IRMOF-6	36.5	298	19.4	Eddaoudi et al., 2002[99]
Zn4O(NDC)3	IRMOF-8	35	298	12.2	Feldblyum et al., 2013[100]

Mg2(dobdc)	Mg-MOF-74, CPO-27-Mg	35	298	13.62	Mason, Veenstra and Long, 2014[87]
Al(OH)(bdc)	MIL-53(Al)	35	298	10.6	Bolinois et al, 2017[101]
Cr(OH)(bdc)	MIL-53(Cr)	30	304	8.8	Bourrelly et al., 2005[102]
Cr3OF(BTC)2	MIL-100(Cr)	35	303	10.7	Llewellyn et al., 2008[103]
Fe3OF(BTC)2	MIL-100(Fe)	35	303	8.8	Wiersum et al., 2013[104]
Cr3OF(H2O)2(bdc)3	MIL-101(Cr)	35	303	9.3	Wiersum et al., 2013[104]
Ti8O8(OH)4(bdc)6	MIL-125	35	303	9.7	Wiersum et al., 2013[104]
Mn2(dobdc)	Mn-MOF-74, CPO-27-Mn	35	297	9.6	Wu, Zhou and Yildirim, 2009[105]
Zn4O(bdc)3	MOF-5 or IRMOF-1	36.5	298	15.1	Eddaoudi et al., 2002[99]
Zn 4O(BTB) 2	MOF -177	35	298	13.8	Furukawa et al., 2010[34]
		65	298	19.5	
Zn 4O(BBC) 2	MOF -200	10	298	5.12	Furukawa et al., 2010[34]
		35	298	11.5	
		65	298	17.5	
Zn 4O(BTB)4/3(NDC)	MOF -205	10	298	5.7	Furukawa et al., 2010[34]
		35	298	14.9	
		65	298	20	
Zn 4O(BTE)4/3(bpdc)	MOF -210	35	298	13.2	Furukawa et al., 2010[34]
		65	298	19.3	
Cu 2(bptc)	MOF -505, NOTT -100	35	300	13.3	He, Zhou, et al., 2013[106]
		65	270	16.2	
Ni 2(dobdc)	Ni -MOF -74, CPO -27 -Ni	35	298	12.2	Mason, Veenstra and Long, 2014[87]
Cu 6(BDPP) 3(H 2O) 6	NJU -Bai10	35	290	17.6	Lu et al., 2013[107]
Cu 2(tptc)	NOTT -101	35	300	17	He, Zhou, et al., 2013[106]
		65	300	19.8	
Cu 2(qptc)	NOTT -102	35	300	18.1	He, Zhou, et al., 2013[106]
		65	300	22.2	
Cu 2(2,6 -nddi)	NOTT -103	35	300	17.9	He, Zhou, et al., 2013[106]

		65	300	20.8	
Cu 2(C26 H20 O 8)	NOTT -107	35	298	15.7	He,Zhou et al., 2014[108]
Cu 2(1,4 -nddi)	NOTT -109	35	300	15.1	He,Zhou et al., 2014[108]
		65	300	18	
Cu 3(C66 H36 O12)	NOTT -119	35	298	16.2	Yan, Yang, et al., 2011[44]
Cu 3 (btdi)(H 2O) 3	NU -111	35	298	19.8	Peng, Srinivas, et al., 2013[109]
		65	270	32.9	
Cu 3(TIPTB)	NU -125, NOTT -122	35	298	18.3	Wilmer et al., 2013[110]
		65	298	22	
Cu 3(TIPTPB)(H 2O) 3	NU -140	65	270	29.6	Barin et al., 2014[111]
		65	300	25.4	
Zr 6 O 4(OH) 4(TPT)	NU -800	35	298	15.2	Gomez -Gualdron et al., 2014[112]
		65	270	27.7	
Cu 2(sbtc)	PCN -11	35	298	14.5	X. -S. Wang, Ma, Rauch, et al., 2008[52]
Cu 2(adip)	PCN -14	65	270	33.3	Peng, Srinivas, et al., 2013[109]
		65	298	26.4	
Cu 2(ebdc)	PCN -16	35	300	15.9	He,Zhou et al., 2014[108]
Cu 2(bdi)	PCN -46	35	298	19.4	Zhao et al., 2010[56]
Cu 3(btei)	PCN -61	35	298	16.7	Yuan et al., 2010[57]
Cu 3(ntei)	PCN -66	35	298	16.6	Yuan et al., 2010[57]
Cu 3(ptei)	PCN -68	35	298	17.9	Yuan et al., 2010[57]
Cu 2(bttcd)	PCN -80	35	296	15	Lu et al., 2012[113]
Zn 2 (TCPBDA)	SNU -30	50	194	6.4	Park, Cheon and Suh, 2010[62]
		50	298	4.5	
Zn 2(TCPBDA)(bpta)	SNU -31	50	298	1.5	Park, Cheon and Suh, 2010[62]
Cu 2(bdcppi)	SNU -50	1	195	10.6	Prasad, Hong and Suh, 2010[64]
		61	298	14.5	
Zn 4O(CVB) 3	SNU -70	1	195	3.8	Prasad and Suh, 2012[114]
		45	298	18.3	

Zn 4O(CEB) 3	SNU -71	1	195	4.7	Prasad and Suh, 2012[114]
		45	298	10.8	
Zn 4O(TCBPA) 2	SNU -77H	1	195	8.04	H. J. Park et al., 2011[66]
		35	298	17.8	
		60	298	18.08	
Zr 6 O 4(OH) 4(bdc) 6	UiO -66	35	303	6.7	Wiersum et al., 2013[104]
Zr6O4(OH)4(NH2-bdc)6	UiO-66-NH2	35	303	7.5	Wiersum et al., 2013[104]
Cu3(BHB)	UTSA-20a	35	298	12.5	He,Zhou et al., 2014[108]
		65	298	14.2	
Cu3(TIPAB)	UTSA-34	35	290	11.9	He,Zhou et al., 2014[108]
Zn4O(NDC)3	UTSA-38	35	300	7.5	Das et al., 2011[115]
Cu2(dceni)	UTSA-40	35	300	12	He, Xiang, et al., 2013[116]
Cu2(PDD)(H2O)2	UTSA-75	65	298	20.4	Li et al., 2015[117]
Cu2(PDDP)(H2O)2	UTSA-76	65	298	20.8	Li et al., 2015[117]
Cu2(tptc)(PDDP)(H2O)2	UTSA-77	65	298	20.4	Li et al., 2015[117]
Cu2(DCPAI)(H2O)2	UTSA-80	65	298	19.4	Wen et al., 2014[118]
Cu2(DFMTP)(H2O)2	UTSA-88	65	298	17.1	Chang et al., 2015[119]
Zn(MeIM)2	ZIF-8	36	300	7	Zhou et al., 2007[72]
Cu2(BDPP)(H2O)2	ZJU-5	65	298	20.8	Li et al., 2015[117]
Cu2(FDDI)	ZJU-25	35	300	17.2	He,Zhou et al., 2014[108]
Cu2(cpda)	ZJU-36	35	300	17	He,Zhou et al., 2014[108]
		65	300	22.7	
Cu6(CTIA)2	ZJU-70	35	298	12.7	Duan et al., 2015[120]
		65	270	16.3	
Cu2(BDEDDI)(H2O)2	ZJUN-50	35	298	17.6	Song, Ling, et al., 2015[121]
		65	298	21.5	
Zn2(BDC)2(dabco)		35	303	12.5	Senkovska and Kaskel, 2008[90]
Zn3O(2,7-ndc)2		35	298	7.3	Park et al., 2009[122]
Zn2(dobdc)	Zn-MOF-74, CPO-27-Zn	35	298	9.1	Wu, Zhou and Yildirim, 2009[105]

Table S3: MOF structures with literature CO_2 uptakes

MOF Name	Common Name	Pressure (Bar)	Temperature (K)	Uptake (%wt)	Reference
Al3(TCPT)6	Alsoc-MOF1	40	298	66.7	Alezi et al., 2015[82]
Zn8(ad)4(BPDC)6O.2Me2NH2	Bio-MOF-1	1	313	5.48	An and Rosi, 2010[123]
Co2(ad)2(CO2CH3)2·2DMF	Bio-MOF-11	1	298	15.3	An, Geib and Rosi, 2010[123]
Al4(OH)2(OCH3)4(NH2-bdc)3	CAU-1	1	273	24.1	Si et al., 2011[124]
Cd(DBNBVP)2(ClO4)2		1	273	12.3	Wu and Lin, 2005[125]
Cd2(HFIDP)(H2O)2		1	195	12.7	Hou et al., 2011[126]
		1	293	8.5	
Cd6(CPOM)3(H2O)6		1	297	5.6	Tian, J et al., 2010[127]
		30	297	14	
Cd(ADA)(4,4′-bipy)0.5.(DMF)	Cd-ADA-1	1	298	3.4	Pachfule et al., 2010[128]
Cd(MeIM)2	CdIF-1	1	273	5.6	Tian,Y.-Q et al., 2010[129]
Cd(eIm)2	CdIF-4	1	273	4	Tian,Y.-Q et al., 2010[129]
Cd(nIm)2	CdIF-9	1	273	8.8	Tian,Y.-Q et al., 2010[129]
Co(BDP)		40	313	41.3	Herm et al., 2011[130]
Co(tImb)		1	298	7.2	Chen, S.-S et al., 2011[131]
Co(tImb).DMF.H2O		1	298	11.7	Chen, S.-S et al., 2011[131]
Co2(dobdc)	Co-MOF-74	0.1	296	10.36	Caskey, Wong-Foy and Matzger, 2008[132]
		1	296	23.6	
Zn(BTT)	CPF-6	1	273	16.2	Lin et al., 2012[133]
Cu2(pmdc)2(bpy)	CPL-2	1	298	6.6	García-Ricard and Hernández Maldonado, 2010[134]
Ni3(OH)(bdc)3(tpt)	CPM-33a	1	273	21.2	Zhao et al., 2015[135]
		1	298	12.7	
Ni3(OH)(dobdc)3(tpt)	CPM-33b	1	273	25.6	Zhao et al., 2015[135]
		1	298	19.8	

(CH3)2NH2][In3O(BTC)2(H2O)3]2[In3(BTC)4]·7DMF·23H2O	CPM-5	1	273	13.7	S.-T. Zheng et al., 2010[136]
		1	299	9.6	
Cu(2-pymo)2		0.86	293	3.8	Navarro et al., 2006[137]
Cu(BDC-OH)		2	296	8.9	Xiang et al., 2012[138]
Cu2(pmdc)2(pz)		1	300	6.4	Kitaura et al., 2005[139]
Cu3(BTB)		20	273	61.1	Zheng et al., 2012[140]
Cu3(BTC)2		1	296	18.8	Xiang et al., 2012[138]
Cu4O(OH)2(Me2trzpba)4		1	298	5.8	Lincke et al., 2011[141]
Cu-BTTri		1	298	12.3	Demessence et al., 2009[142]
		40	313	42.8	
Cu3(TDPAT)(H2O)3	Cu-TDPAT	0.1	298	5.8	Li et al., 2012[91]
		1	298	20.6	
Cu3(TPBTM)(H2O)3	Cu-TPBTM	20	298	50.8	Baishu Zheng et al., 2011[143]
Fe3[(Fe4Cl)3(BTT)8(MeOH)4]2	Fe-BTT	1	298	12	Sumida et al., 2010[144]
Fe3O(OH)(BTC)2	Fe-MIL-100	0.9	298	6.6	Mason et al., 2015[145]
Fe(pz)Ni(CN)4		1	298	9.3	Sumida et al 2012[146]
Cu(BTTA)(H2O)	FJI-H14	1	195	35.5	Liang et al., 2017[147]
		1	298	22.2	
Zn(bcphfp)	FMOF-2	1	298	4.5	Fernandez et al., 2010[148]
Cu3(BTC)2	HKUST-1	1	298	15.3	Millward and Yaghi, 2005[149]
		30	298	31.9	
[Zn3(pbdc)2]·HPIP·H3O·5H2O	HPIP@ZnPC -2	0.15	298	4.6	Ling et al., 2013[150]
[Zn3(pbdc)2]·HPYR·H3O·4H2O	HPYR@ZnP C-2	0.15	298	3	Ling et al., 2013[150]
Zn(BHTHT)	IFMC-1	1	195	24.8	Qin et al., 2012[151]
		1	273	15.3	
		1	298	10.6	
Zn(Mlai)	IMOF-3	1	273	7.2	Debatin et al., 2010[81]
In2(OH)2(obb)2		1	298	8.6	Y.-X. Tan et al., 2011[152]
In3O(abtc)1.5(H2O)3(NO3)	socMOF M080	1	273	17.9	Moellmer et al., 2010[153]

In3(BTC)4(choline)		1	273	12.3	Chen et al., 2009[154]
Zn4O(NH2-BDC)2	IRMOF-3	1.1	298	5.1	Millward and Yaghi, 2005[149]
		35	298	45.1	
Zn4O(dobdc)2	IRMOF-6	1.2	298	4.6	Millward and Yaghi, 2005[149]
		40	298	46.6	
Zn4O(NDC)3	IRMOF-8	1	273	12.2	Orefuwa et al., 2013[155]
		30	273	38.6	
		30	298	34	
Zn4O(HPDC)2	IRMOF-11	1.1	298	7.3	Millward and Yaghi, 2005[149]
		35	298	39.3	
Zn4O(TPDC)3	IRMOF-16	1	298	5.4	Bae et al., 2009[156]
Mg2(DH3PhDC)-CH2NH2	IRMOF-74- III-CH2NH2	1	298	12.3	Fracaroli et al., 2014[157]
Fe3O(TCDC)(H2O)3	LIFM-26	1	273	19.2	C.-X Chen et al., 2017[158]
		1	298	12.7	
Zr6O8(TDA)(H2O)8	LIFM-29	1	273	9.9	C.-X. Chen et al., 2016[159]
		1	298	6.13	
Zr6O8(tftpa)(H2O)8	LIFM-30	1	273	10.3	C.-X. Chen et al., 2016[159]
		1	298	4.9	
Zr6O8(NDC)(H2O)8	LIFM-31	1	273	10.3	C.-X. Chen et al., 2016[159]
		1	298	6.5	
Zr6O8(dhbpdc)(H2O)8	LIFM-32	1	273	10.6	C.-X. Chen et al., 2016[159]
		1	298	5.25	
Zr6O8(dabpdc)(H2O)8	LIFM-33	1	273	13.7	C.-X. Chen et al., 2016[159]
		1	298	7.08	
Cu(etz)	MAF-2	1	298	3.6	Zhang and Chen, 2009[160]
		1	273	8.84	
Zn2(btm)2	MAF-23	1	273	12.7	Liao et al., 2012[161]
		1	298	9.9	

Co(dpt24)2	MAF-25	1	195	18.6	Lin, Zhang and Chen, 2010[162]
		1	273	4.83	
		1	283	3	
Co(mdpt24)2	MAF-26	1	195	14.3	Lin, Zhang and Chen, 2010[162]
		1	273	3.75	
		1	283	2.9	
Zn(atz)2	MAF-66	1	273	21.7	Lin et al., 2012[163]
		1	298	16.2	
Mg(TCPBDA)		1	195	20.9	Cheon et al 2009[164]
		1	273	8.3	
		1	298	6.2	
Mg2(dobpdc)		0.15	298	12.1	McDonald et al., 2012[165]
		1	298	14.5	
Mg(3,5-pdc)	Mg-MOF-1	1	298	2.7	Mallick et al., 2010[166]
Mg2(dobdc)	Mg-MOF74, MgCPO-27	0.1	298	20.3	Bao et al., 2011[167]
		1	298	27.5	Dietzel, Besikiotis and Blom, 2009[168]
		36	278	40.8	
Al(OH)(bdc)	MIL-53(Al), USO-1-Al	1	303	9.2	Rallapalli et al., 2011[169]
		25	304	30.6	Bourrelly et al., 2005[102]
Cr(OH)(bdc)	MIL-53(Cr	1	304	8.1	Bourrelly et al., 2005[102]
		25	304	30.8	
Al12O(OH)18(Al2(OH)4)(BTC)6	MIL-96(Al)	10	303	16.2	Loiseau et al., 2006[170]
		20	303	18.6	
Cr3OF(H2O)3(BTC)2	MIL-100(Cr)	50	304	44.2	Llewellyn et al., 2008[103]
Cr3OF(H2O)2(BDC)3	MIL-101(Cr)	1	296	5.8	Chowdhury et al., 2012[171]
		50	304	56.9	Llewellyn et al., 2008[103]
Cr3OF(H2O)2(NTC)1.5	MIL-102(Cr)	30	304	12	Surblé et al., 2006[172]
Al4(OH)8(C10O8H2)	MIL-120	10	303	15.6	Volkringer et al., 2009[173]
mmen-Cu-BTTri		1	298	15.6	McDonald et al., 2011[174]

Mn(bdc)(dpe)		1	195	17.7	Foo et al., 2016[175]
Mn(NDC)(DEF)		1	195	11.7	Hoi, Kobayashi and Myunghyun,. 2006[176]
		1	273	6.2	
Mn(pmdc)		0.9	293	7.3	Beobide et al., 2008[177]
[Mn2(2,6-ndc)2(bpda)2]·5DMF	Mn-bpda	1	195	24.5	Lee et al., 2016[178]
		35	298	21.6	
Zn3(bdc)3	MOF-2	1	298	2.4	Millward and Yaghi, 2005[149]
		30	298	11.97	
Zn4O(bdc)3	MOF-5 or IRMOF-1	1	296	3.94	Millward and Yaghi, 2005[149]
		35	298	48.8	
Zn4O(BTB)2	MOF-177	1	298	6.5	Saha et al., 2010[179]
		50	298	60.8	Furukawa et al., 2010[34]
Zn4O(BBC)2(H2O)3	MOF-200	50	298	73.9	Furukawa et al., 2010[34]
Zn4O(BTB)(NDC)(H2O)3	MOF-205	50	298	62.6	Furukawa et al., 2010[34]
Zn4O(BTE)(BPDC)(H2O)3	MOF-210	50	298	74.2	Furukawa et al., 2010[34]
Al(OH)(bpydc	MOF-253	1	298	6.2	Bloch et al., 2010[177]
Cu2(bptc)(H2O)2(DMF)3	MOF-505	1.1	298	12.6	Millward and Yaghi, 2005[149]
Zn2(BDC)2(4,4'-bpy)	MOF-508	5	303	20.6	Bárcia et al., 2008[180]
Al(ABDC)(OH)	NH2-MIL53(Al)	5	303	8.4	Couck et al., 2009[181]
		13	303	22.8	
Ti(NH2-bdc)2	NH2-MIL125	1	273	15	Kim et al., 2013[63]
		1	298	8.8	
Ni(DBNBVP)2Cl2		1	273	8.1	Wu and Lin, 2006[182]
Ni3(L-TMTA)2(bpy)4		1	298	8.2	Z. Chen et al., 2011[183]
Ni-4Pyc		10	298	26.5	Nandi et al., 2015[184]
Ni2(dobdc)	Ni-MOF-74, CPO-27-N	0.1	296	10.3	Caskey, Wong-Foy and Matzger, 2008[132]
		1	296	20.3	
		22	278	34.4	Dietzel, Besikiotis and Blom, 2009[168]

Cu3(cobai)2(H2O)5	NJU-Bai3	1	273	21.5	Duan et al., 2012[185]
		20	273	49.3	
		20	298	44.3	
[Cu2(H2O)2(obaddi)]·4 H2O·2 DMA	NOTT-125	1	273	28.6	Alsmail et al., 2014[186]
		1	298	15.4	
		20	273	50.3	
		20	298	48.3	
Cu4(TDTM)	NOTT-140	1	293	11.7	C. Tan et al., 2011[45]
		20	293	47.7	
		20	283	46.2	
Me2NH2)1.75[In(bptpc)]1.75(DMF)12	NOTT-202a	1	195	46.8	Yang et al., 2012[187]
Cu3(btdi)(H2O)3	NU-111	30	298	62.8	Peng, Srinivas, et al., 2013[109]
Zr(TBAPy)2	NU-1000	1	298	10.7	Farha et al., 2010[47]
		40	298	67.1	
Cu3(TATB)2 (catenated)	PCN-6	1	273	25.65	Kim et al., 2011[63]
		1	298	15.9	
		30	298	53.9	
Cu3(btei)	PCN-61	35	298	50.8	Yuan et al., 2010[57]
Cu3(ntei)	PCN-66	35	298	53.6	Yuan et al., 2010[57]
Cu3(ptei)	PCN-68	35	298	57.2	Yuan et al., 2010[57]
Cu(NddIP)0.5	PCN-88	1	273	23.8	Li et al., 2013[188]
		1	296	15.6	
Zn(PDAT)	PCN-123	1	295	3.9	J. Park et al., 2012[189]
Zr(TCPP)2	PCN-222, MOF-545	1	298	4.9	Lv et al., 2018[190]
		30	298	37.6	
Pd(2-pymo)2		0.86	293	6.8	Navarro et al., 2006[137]
Pd(F-pymo)2		0.86	293	9.7	Navarro et al., 2007[191]
		1.2	273	13.5	
Sc2(BDC)3		1	303	3	Miller et al., 2009[192]
(Me2NH2)[In(ABDC)2]	SHF-61	20	298	9.6	Carrington et al., 2017[193]

Zn3(NTB)2	SNU-3	1	195	22.9	Suh, Cheon and Lee, 2007[194]
		1	273	6.5	
Zn2(abtc)(DMF)2	SNU-4	1	195	35.5	Lee et al., 2008[61]
		1	273	17.3	
Cu2(abtc)3	SNU-5	1	195	52.9	Lee et al., 2008[61]
		1	273	27.8	
Cu2(BPnDC)2(4,4’-bpy)	SNU-6	1	273	9.9	Park and Suh, 2008[195]
Zn2(BPnDC)2(bpy)	SNU-9	30	298	23	Park and Suh, 2010[196]
Co2(MTB)	SNU-15	1	273	6.5	Cheon and Suh, 2009[197]
Cu2(TCM)	SNU-21S	1	298	10	Kim and Suh, 2011[63]
Zn2(TCPBDA)(H2O)2	SNU-30	1	195	31.6	Park, Cheon and Suh, 2010[62]
		1	273	10.3	
		1	298	4.9	
		50	298	18	
Zn2(TCPBDA)(bpta)	SNU-31	1	195	14.6	Park, Cheon and Suh, 2010[62]
		1	273	5	
		1	298	2.6	
		40	298	8.8	
Cu2(bdcppi)	SNU-50	1	195	47.2	Prasad, Hong and Suh, 2010[64]
		1	273	19.1	
		1	298	13.7	
		46	298	42.9	
Zn4O(CEB)2	SNU-71	1	195	36.7	Prasad and Suh, 2012[114]
		1	298	4.2	
Ni2(CYC-2C-CYC)(bptc)	SNU-M10	1	195	19.5	Choi and Suh, 2009[198]
		1	273	12.7	
		1	298	8.5	
Ni2(CYC-4C-CYC)(bptc)	SNU-M11	1	195	19.5	Choi and Suh, 2009[198]
[Zn4O(bdc)3].(ZnO)0.125	SUMOF-2	1	273	15.9	Yao et al., 2012[199]
Zn4O(NDC)3	SUMOF-3	1	273	13	Yao et al., 2012[199]

Zn4O(bdc)2(bpdc)(H2O)	SUMOF-4	1	273	13.7	Yao et al., 2012[199]
Cu(bpy)2(EDS)	TMOF-1	1	200	22.9	Zhang et al., 2016[200]
		1	273	9	
		1	298	5.8	
		1	308	4.9	
Zn2(obb)2(bpta)	TMU-22	1	203	24.1	Safarifard et al., 2016[201]
Zn2(obb)2(bpfn)	TMU-24	1	203	21.7	Safarifard et al., 2016[201]
Zr6O4(OH)4(bpdc)12	UiO(bpdc)	20	303	42.1	Li et al., 2014[202]
Zn4O(BDC)(BTB)4/3	UMCM-1	1	298	3.8	Yazaydın et al., 2009[203]
		24	298	50.8	Mu, Schoenecker and Walton, 2010[204]
Cu3(BPT)2	UMCM-150	1	298	10.2	Yazaydın et al., 2009[203]
Ni2(BDC)2(DABCO)	USO-2-Ni	1	298	10	Arstad et al., 2008[205]
Ni2(2-amino-BDC)2(DABCO)	USO-2-Ni-A	1	298	14	Arstad et al., 2008[205]
In(OH)(BDC)	USO-3-In-A	1	298	8	Arstad et al., 2008[205]
Al(OH)(Sbpdc)	USTC-253	1	273	14	Jiang et al., 2015[206]
		1	298	8.5	
Al(OH)(Sbpdc)(TFA)	USTC-253- TFA	1	273	21.2	Jiang et al., 2015[206]
		1	298	11.3	
Cu(BDC−OH)(4,4′-bipy)	UTSA-15a	1	296	5.1	Z. Chen, Xiang, Hadi D. Arman, et al., 2011a[207]
Cu3(BHB)	UTSA-20a	1	296	17	Guo et al., 2011[69]
Zn(BDC-OH)(DABCO)0.5	UTSA-25a	1	296	12	Z. Chen, Xiang, Hadi D Arman, et al., 2011b[208]
Zn4(TIPAB)(DMA)4	UTSA-33a	1	273	8.9	He, Zhang, Xiang, Fronczek, et al., 2012[209]
Zn2(bttb)(diPyNI)	YO-MOF	1	273	14.4	Mulfort et al., 2010[210]
		1	298	4.5	
Zn(MeIM)2	ZIF-8	1	298	4.3	Yazaydın et al., 2009[203]
Zn(nIm)(bIm)	ZIF-68	1	298	6.4	Banerjee et al., 2009[211]
Zn(nIm)(cbIm)	ZIF-69	1	298	7.9	Banerjee et al., 2009[211]
Zn(nIm)(Im)	ZIF-70	1	298	4.43	Banerjee et al., 2009[211]
Zn(nbIm)(nIm)	ZIF-78	2	298	9.52	Banerjee et al., 2009[211]

Zn(nIm)(mbIm)	ZIF-79	1.1	298	5.9	Banerjee et al., 2009[211]
Zn(nIm)(cnIm)	ZIF-82	1	298	7.84	Banerjee et al., 2009[211]
Zn(almeIm)	ZIF-93	1	298	6.7	Morris et al., 2010[212]
Zn(cyamIm)	ZIF-96	1	298	8.5	Morris et al., 2010[212]
Cu2(BTADD)(H2O)2	ZJNU-40	1	273	24.9	Song et al., 2014[213]
		1	296	16.3	
[Cu2(qdip)(H2O)2]·3DMF·2EtOH	ZJNU-43	1	273	24.9	Song, Hu, et al., 2015[214]
		1	296	17.2	
[Cu2(qodip)(H2O)2]·3DMF·3MeOH Z	ZJNU-45	1	273	23.5	Song, Hu, et al., 2015[214]
		1	296	16.1	
Zn(BDC-NH2)(dabco)0.5		1	298	8.8	Zhao et al., 2011[215]
Zn(BDC-OH)(dabco)0.5		1	298	11.7	Zhao et al., 2011[215]
Zn(BPZNO2)		1.2	298	18	Mosca et al., 2018[216]
Zn(dtp)		1	195	14	J.-R. Li et al., 2008[217]
Zn(MIai)		1	298	7.9	Debatin et al., 2010[81]
Zn2(2,5-BME-bdc)2(dabco)		1	195	16.8	Henke et al., 2012[218]
Zn2(BDC)2(dabco)		1	294	8.1	Mishra et al., 2012[219]
		25	294	37.3	
Zn2(BME-bdc)2-(bipy)		1	195	23.6	Henke and Fischer, 2011[220]
		1	273	9.2	
		1	298	5.56	
Zn2(BMOE-bdc)2(dabco)		1	195	25.4	Henke et al., 2010[221]
Zn2(BMOP-bdc)2(dabco)		1	195	8.6	Henke et al., 2010[221]
Zn2(bpdc)2(bpe)		1	273	7.3	J. Zhang et al., 2010[222]
		1	298	4.7	
Zn2(DB-bdc)2(dabco)		1	195	9.9	Henke et al., 2012[218]
Zn2(MOE-bdc)2(dabco)		1	195	30.7	Henke et al., 2010[221]
Zn2(NDC)2(diPyNI)		1	298	5.8	Bae et al., 2011[223]
Zn2(ox)(atz)2		1.2	273	16.1	Vaidhyanathan et al., 2009[224]
		1.2	293	14.2	

Zn4(OH)2(BTC)2		1	295	6.7	Z. Zhang et al., 2010[225]
Zn4O(BMOE-bdc)3		1	195	18.1	Henke et al., 2010[221]
Zn4O(BMOP-bdc)3		1	195	11.9	Henke et al., 2010[221]
Zn4O(FMA)3		2	296	3.8	Xiang et al., 2012[138]
Zn4O(MOE-bdc)3		1	195	47	Henke et al., 2010[221]
[Zn4(BDC)4(BPDA)4]·5DMF·3H2O	Zn-bpda	1	298	7.5	Lee et al., 2013[226]
		35	298	13.2	
Zn2(dobdc)	Zn-MOF-74	0.1	296	5.4	Caskey, Wong-Foy and Matzger, 2008[132]
		1	296	19.5	
Zn(5-AT)2	ZTF-1	1	273	19.8	Panda et al., 2011[80]

Table S4: Individual coefficient of determination values for each fold in the 10 runs

	Fold number									
Run	1	2	3	4	5	6	7	8	9	10
1	0.91	0.87	0.87	0.92	0.87	0.87	0.92	0.86	0.90	0.85
2	0.86	0.88	0.89	0.90	0.84	0.86	0.86	0.92	0.84	0.92
3	0.86	0.86	0.93	0.91	0.90	0.83	0.92	0.90	0.87	0.83
4	0.85	0.87	0.89	0.91	0.92	0.82	0.87	0.87	0.89	0.88
5	0.89	0.90	0.93	0.83	0.89	0.88	0.87	0.85	0.84	0.92
6	0.89	0.90	0.82	0.94	0.86	0.89	0.86	0.87	0.89	0.85
7	0.88	0.87	0.87	0.91	0.91	0.89	0.79	0.86	0.90	0.87
8	0.89	0.84	0.92	0.86	0.88	0.88	0.88	0.85	0.86	0.88
9	0.79	0.91	0.87	0.87	0.92	0.90	0.82	0.91	0.90	0.86
10	0.88	0.89	0.83	0.81	0.88	0.88	0.87	0.89	0.91	0.91

Hyperparameters for other machine learning models

Linear Regression

This was fitted using the "fitrlinear" function in MATLAB 2020. All other parameters that are not stated are set to their default values.

Lambda = 0.048818

Learner type = Least Squares

Regularisation = Ridge

Decision Trees

This was fitted using the "fitrtrees" function in MATLAB 2020. All other parameters that are not stated are set at their default values.

Minimum Leaf size = 3

Maximum number of splits = 195

Support Vector Mechanism

This was fitted using the "fitrsvm" function in MATLAB 2020. All other parameters that are not stated are set at their default values.

Box Constraint = 0.11404

Epsilion = 0.18408

Kernel Function = Linear

Standardize data = False

References for uptake data

(1) Sumida, K.; Hill, M. R.; Horike, S.; Dailly, A.; Long, J. R. Synthesis and Hydrogen Storage Properties of Be12 (OH) 12 (1, 3, 5-Benzenetribenzoate) 4. *J. Am. Chem. Soc.* **2009**, *131*, 15120–15121.

(2) Chun, H.; Kim, D.; Dybtsev, D. N.; Kim, K. Metal--Organic Replica of Fluorite Built with an Eight-Connecting Tetranuclear Cadmium Cluster and a Tetrahedral Four-Connecting Ligand. *Angew. Chemie* **2004**, *116*, 989–992.

(3) Choi, H. J.; Dinca, M.; Long, J. R. Broadly Hysteretic H2 Adsorption in the Microporous Metal- Organic Framework Co (1, 4-Benzenedipyrazolate). *J. Am. Chem. Soc.* **2008**, *130*, 7848–7850.

(4) Li, Y.; Xie, L.; Liu, Y.; Yang, R.; Li, X. Favorable Hydrogen Storage Properties of M(HBTC)(4,4′-Bipy) ·3DMF (M = Ni and Co). *Inorg. Chem.* **2008**, *47*, 10372–10377. https://doi.org/10.1021/ic800919k.

(5) Myunghyun Paik Suh, Hye Jeong Park, T. K. P. Hydrogen Storage in Metal Organic Frameworks. *Chem. Rev.* **2012**, *112*, 782–835.

(6) Lee, J. Y.; Pan, L.; Kelly, S. P.; Jagiello, J.; Emge, T. J.; Li, J. Achieving High Density of Adsorbed Hydrogen in Microporous Metal Organic Frameworks. *Adv. Mater.* **2005**, *17*, 2703–2706. https://doi.org/10.1002/adma.200500867.

(7) Chun, H.; Jung, H.; Koo, G.; Jeong, H.; Kim, D.-K. Efficient Hydrogen Sorption in 8-Connected MOFs Based on Trinuclear Pinwheel Motifs. *Inorg. Chem.* **2008**, *47*, 5355–5359.

(8) Liao, J. H.; Chen, W. T.; Tsai, C. S.; Wang, C. C. Characterization, Adsorption Properties, Metal Ion-Exchange and Crystal-to-Crystal Transformation of Cd3[(Cd4Cl) 3(BTT)8(H2O)12]2 Framework, Where BTT3- = 1,3,5-Benzenetristetrazolate. *CrystEngComm* **2013**, *15*, 3377–3384. https://doi.org/10.1039/c3ce27063g.

(9) Sumida, K.; Her, J.-H.; Dinca, M.; Murray, L. J.; Schloss, J. M.; Pierce, C. J.; Thompson, B. A.; FitzGerald, S. A.; Brown, C. M.; Long, J. R. Neutron Scattering and Spectroscopic Studies of Hydrogen Adsorption in Cr3 (BTC) 2□ A Metal- Organic Framework with Exposed Cr2+ Sites. *J. Phys. Chem. C* **2011**, *115*, 8414–8421.

(10) Yang, W.; Lin, X.; Jia, J.; Blake, A. J.; Wilson, C.; Hubberstey, P.; Champness, N. R.; Schröder, M. A Biporous Coordination Framework with High H 2 Storage Density. *Chem. Commun.* **2008**, 359–361.

(11) Liu, X.; Oh, M.; Lah, M. S. Adsorbate Selectivity of Isoreticular Microporous Metal--Organic Frameworks with Similar Static Pore Dimensions. *Cryst. Growth Des.* **2011**, *11*, 5064–5071.

(12) Nouar, F.; Eubank, J. F.; Bousquet, T.; Wojtas, L.; Zaworotko, M. J.; Eddaoudi, M. Supermolecular Building Blocks (SBBs) for the Design and Synthesis of Highly Porous Metal-Organic Frameworks. *J. Am. Chem. Soc.* **2008**, *130*, 1833–1835. https://doi.org/10.1021/ja710123s.

(13) Lee, J. Y.; Olson, D. H.; Pan, L.; Emge, T. J.; Li, J. Microporous Metal-Organic Frameworks with High Gas Sorption and Separation Capacity. *Adv. Funct. Mater.* **2007**, *17*, 1255–1262. https://doi.org/10.1002/adfm.200600944.

(14) Zheng, B.; Liang, Z.; Li, G.; Huo, Q.; Liu, Y. Synthesis, Structure, and Gas Sorption Studies of a Three-Dimensional Metal-Organic Framework with NbO Topology. *Cryst. Growth Des.* **2010**, *10*, 3405–3409. https://doi.org/10.1021/cg100046j.

(15) García-Holley, P.; Schweitzer, B.; Islamoglu, T.; Liu, Y.; Lin, L.; Rodriguez, S.; Weston, M. H.; Hupp, J. T.; Gómez-Gualdrón, D. A.; Yildirim, T.; Farha, O. K. Benchmark Study of Hydrogen Storage in Metal-Organic Frameworks under Temperature and Pressure Swing Conditions. *ACS Energy Lett.* **2018**, *3*, 748–754. https://doi.org/10.1021/acsenergylett.8b00154.

(16) Maity, K.; Karan, C. K.; Biradha, K. Porous Metal-Organic Polyhedral Framework Containing Cuboctahedron Cages as SBUs with High Affinity for H2 and CO2 Sorption: A Heterogeneous Catalyst for Chemical Fixation of CO2. *Chem. - A Eur. J.* **2018**, *24*, 10988–10993. https://doi.org/10.1002/chem.201802829.

(17) Jia, J.; Lin, X.; Wilson, C.; Blake, A. J.; Champness, N. R.; Hubberstey, P.; Walker, G.; Cussen, E. J.; Schröder, M. Twelve-Connected Porous Metal-Organic Frameworks with High H2 Adsorption. *Chem. Commun.* **2007**, *3*, 840–842. https://doi.org/10.1039/b614254k.

(18) Choi, S. B.; Seo, M. J.; Cho, M.; Kim, Y.; Jin, M. K.; Jung, D. Y.; Choi, J. S.; Ahn, W. S.; Rowsell, J. L. C.; Kim, J. A Porous and Interpenetrated Metal-Organic Framework Comprising Tetranuclear Iron III-Oxo Clusters and Tripodal Organic Carboxylates and Its Implications for (3,8)-Coordinated Networks. *Cryst. Growth Des.* **2007**, *7*, 2290–2293. https://doi.org/10.1021/cg070640e.

(19) Han, D.; Jiang, F. L.; Wu, M. Y.; Chen, L.; Chen, Q. H.; Hong, M. C. A Non-Interpenetrated Porous Metal-Organic Framework with High Gas-Uptake Capacity. *Chem. Commun.* **2011**, *47*, 9861–9863. https://doi.org/10.1039/c1cc12858b.

(20) Dincă, M.; Han, W. S.; Liu, Y.; Dailly, A.; Brown, C. M.; Long, J. R. Observation of Cu2+-H2 Interactions in a Fully Desolvated Socialite-Type Metal-Organic Framework. *Angew. Chemie - Int. Ed.* **2007**, *46*, 1419–1422. https://doi.org/10.1002/anie.200604362.

(21) Cheng, C.; Yang, G. S.; Yan, L. H.; Wang, X.; Jiang, C. J.; Li, N.; Su, Z. M. Improved Moisture Resistance of a New Zn4O-Based Metal-Organic Framework by Introducing Hydrophobic Phenyl Groups. *Inorg. Chem. Commun.* **2018**, *93*, 25–28. https://doi.org/10.1016/j.inoche.2018.04.026.

(22) Wong-Foy, A. G.; Matzger, A. J.; Yaghi, O. M. Exceptional H2 Saturation Uptake in Microporous Metal-Organic Frameworks. *J. Am. Chem. Soc.* **2006**, *128*, 3494–3495. https://doi.org/10.1021/ja058213h.

(23) Rowsell, J. L. C.; Millward, A. R.; Park, K. S.; Yaghi, O. M. Hydrogen Sorption in Functionalized Metal-Organic Frameworks. *J. Am. Chem. Soc.* **2004**, *126*, 5666–5667. https://doi.org/10.1021/ja049408c.

(24) Rowsell, J. L. C.; Yaghi, O. M. Effects of Functionalization, Catenation, and Variation of the Metal Oxide and Organic Linking Units on the Low-Pressure Hydrogen Adsorption Properties of Metal-Organic Frameworks. *J. Am. Chem. Soc.* **2006**, *128*, 1304–1315.

https://doi.org/10.1021/ja056639q.

(25) Fang, Q.-R.; Zhu, G.-S.; Jin, Z.; Ji, Y.-Y.; Ye, J.-W.; Xue, M.; Yang, H.; Wang, Y.; Qiu, S.-L. Mesoporous Metal–Organic Framework with Rare Etb Topology for Hydrogen Storage and Dye Assembly. *Angew. Chemie* **2007**, *119*, 6758–6762. https://doi.org/10.1002/ange.200700537.

(26) Yan, Y.; Da Silva, I.; Blake, A. J.; Dailly, A.; Manuel, P.; Yang, S.; Schröder, M. High Volumetric Hydrogen Adsorption in a Porous Anthracene-Decorated Metal-Organic Framework. *Inorg. Chem.* **2018**, *57*, 12050–12055. https://doi.org/10.1021/acs.inorgchem.8b01607.

(27) Sumida, K.; Brown, C. M.; Herm, Z. R.; Chavan, S.; Bordiga, S.; Long, J. R. Hydrogen Storage Properties and Neutron Scattering Studies of Mg2(Dobdc)—a Metal-Organic Framework with Open Mg2+ Adsorption Sites. *Chem. Commun.* **2011**, *47*, 1157–1159. https://doi.org/10.1039/c0cc03453c.

(28) Férey, G.; Latroche, M.; Serre, C.; Millange, F.; Loiseau, T.; Percheron-Guégan, A. Hydrogen Adsorption in the Nanoporous Metal-Benzenedicarboxylate M(OH)(O2C-C6H4-CO2)(M = Al3+, Cr3+), MIL-53. *Chem. Commun.* **2003**, *3*, 2976–2977. https://doi.org/10.1039/b308903g.

(29) Latroche, M.; Surblé, S.; Serre, C.; Mellot-Draznieks, C.; Llewellyn, P. L.; Lee, J.-H.; Chang, J.-S.; Jhung, S. H.; Férey, G. Hydrogen Storage in the Giant-Pore Metal–Organic Frameworks MIL-100 and MIL-101. *Angew. Chemie* **2006**, *118*, 8407–8411. https://doi.org/10.1002/ange.200600105.

(30) Ren, J.; Musyoka, N. M.; Langmi, H. W.; Segakweng, T.; North, B. C.; Mathe, M.; Kang, X. Modulated Synthesis of Chromium-Based Metal-Organic Framework (MIL-101) with Enhanced Hydrogen Uptake. *Int. J. Hydrogen Energy* **2014**, *39*, 12018–12023. https://doi.org/10.1016/j.ijhydene.2014.06.019.

(31) Panella, B.; Hirscher, M.; Pütter, H.; Müller, U. Hydrogen Adsorption in Metal--Organic Frameworks: Cu-MOFs and Zn-MOFs Compared. *Adv. Funct. Mater.* **2006**, *16*, 520–524.

(32) Sabo, M.; Henschel, A.; Fröde, H.; Klemm, E.; Kaskel, S. Solution Infiltration of Palladium into MOF-5: Synthesis, Physisorption and Catalytic Properties. *J. Mater. Chem.* **2007**, *17*, 3827–3832. https://doi.org/10.1039/b706432b.

(33) Liu, Y.; Kabbour, H.; Brown, C. M.; Neumann, D. A.; Ahn, C. C. Increasing the Density of Adsorbed Hydrogen with Coordinatively Unsaturated Metal Centers in Metal-Organic Frameworks. *Langmuir* **2008**, *24*, 4772–4777. https://doi.org/10.1021/la703864a.

(34) Furukawa, H.; Ko, N.; Go, Y. B.; Aratani, N.; Choi, S. B.; Choi, E.; Yazaydin, A. Ö.; Snurr, R. Q.; O'Keeffe, M.; Kim, J.; Yaghi, O. M. Ultrahigh Porosity in Metal-Organic Frameworks. *Science (80-.).* **2010**, *329*, 424–428. https://doi.org/10.1126/science.1192160.

(35) Chen, B.; Ockwig, N. W.; Millward, A. R.; Contreras, D. S.; Yaghi, O. M. High H2 Adsorption in a Microporous Metal-Organic Framework with Open Metal Sites. *Angew. Chemie - Int. Ed.* **2005**, *44*, 4745–4749. https://doi.org/10.1002/anie.200462787.

(36) Lin, X.; Telepeni, I.; Blake, A. J.; Dailly, A.; Brown, C. M.; Simmons, J. M.; Zoppi, M.; Walker, G. S.; Thomas, K. M.; Mays, T. J.; Hubberstey, P.;

Champness, N. R.; Schröder, M. High Capacity Hydrogen Adsorption in Cu(II) Tetracarboxylate Framework Materials: The Role of Pore Size, Ligand Functionalization, and Exposed Metal Sites. *J. Am. Chem. Soc.* **2009**, *131*, 2159–2171. https://doi.org/10.1021/ja806624j.

(37) Barman, S.; Furukawa, H.; Blacque, O.; Venkatesan, K.; Yaghi, O. M.; Berke, H. Azulene Based Metal-Organic Frameworks for Strong Adsorption of H 2. *Chem. Commun.* **2010**, *46*, 7981–7983. https://doi.org/10.1039/c0cc02589e.

(38) Xia, L.; Liu, Q.; Wang, F.; Lu, J. Improving the Hydrogen Storage Properties of Metal-Organic Framework by Functionalization. *J. Mol. Model.* **2016**, *22*. https://doi.org/10.1007/s00894-016-3129-3.

(39) Dietzel, P. D. C.; Panella, B.; Hirscher, M.; Blom, R.; Fjellvåg, H. Hydrogen Adsorption in a Nickel Based Coordination Polymer with Open Metal Sites in the Cylindrical Cavities of the Desolvated Framework. *Chem. Commun.* **2006**, *1*, 959–961. https://doi.org/10.1039/b515434k.

(40) Zheng, B.; Yun, R.; Bai, J.; Lu, Z.; Du, L.; Li, Y. Expanded Porous MOF-505 Analogue Exhibiting Large Hydrogen Storage Capacity and Selective Carbon Dioxide Adsorption. *Inorg. Chem.* **2013**, *52*, 2823–2829. https://doi.org/10.1021/ic301598n.

(41) Yang, S.; Lin, X.; Dailly, A.; Blake, A. J.; Hubberstey, P.; Champness, N. R.; Schröder, M. Enhancement of H2 Adsorption in Coordination Framework Materials by Use of Ligand Curvature. *Chem. - A Eur. J.* **2009**, *15*, 4829–4835. https://doi.org/10.1002/chem.200802292.

(42) Yan, Y.; Lin, X.; Yang, S.; Blake, A. J.; Dailly, A.; Champness, N. R.; Hubberstey, P.; Schröder, M. Exceptionally High H2 Storage by a Metal-Organic Polyhedral Framework. *Chem. Commun.* **2009**, 1025–1027. https://doi.org/10.1039/b900013e.

(43) Yan, Y.; Blake, A. J.; Lewis, W.; Barnett, S. A.; Dailly, A.; Champness, N. R.; Schröder, M. Modifying Cage Structures in Metal-Organic Polyhedral Frameworks for H 2 Storage. *Chem. - A Eur. J.* **2011**, *17*, 11162–11170. https://doi.org/10.1002/chem.201101341.

(44) Yan, Y.; Yang, S.; Blake, A. J.; Lewis, W.; Poirier, E.; Barnett, S. A.; Champness, N. R.; Schröder, M. A Mesoporous Metal-Organic Framework Constructed from a Nanosized C 3-Symmetric Linker and [Cu24(Isophthalate)24] Cuboctahedra. *Chem. Commun.* **2011**, *47*, 9995–9997. https://doi.org/10.1039/c1cc13170b.

(45) Tan, C.; Yang, S.; Champness, N. R.; Lin, X.; Blake, A. J.; Lewis, W.; Schröder, M. High Capacity Gas Storage by a 4,8-Connected Metal-Organic Polyhedral Framework. *Chem. Commun.* **2011**, *47*, 4487–4489. https://doi.org/10.1039/c1cc10378d.

(46) Ibarra, I. A.; Yang, S.; Lin, X.; Blake, A. J.; Rizkallah, P. J.; Nowell, H.; Allan, D. R.; Champness, N. R.; Hubberstey, P.; Schröder, M. Highly Porous and Robust Scandium-Based Metal-Organic Frameworks for Hydrogen Storage. *Chem. Commun.* **2011**, *47*, 8304–8306. https://doi.org/10.1039/c1cc11168j.

(47) Farha, O. K.; Yazaydin, A. Ö.; Eryazici, I.; Malliakas, C. D.; Hauser, B. G.; Kanatzidis, M. G.; Nguyen, S. T.; Snurr, R. Q.; Hupp, J. T. De Novo Synthesis of a Metal-Organic Framework Material Featuring Ultrahigh Surface Area and Gas Storage Capacities. *Nat. Chem.* **2010**, *2*, 944–948. https://doi.org/10.1038/nchem.834.

(48) Yan, Y.; Yang, S.; Blake, A. J.; Schröder, M. Studies on Metal-Organic Frameworks of Cu(II) with Isophthalate Linkers for Hydrogen Storage. *Acc. Chem. Res.* **2014**, *47*, 296–307. https://doi.org/10.1021/ar400049h.

(49) Gómez-Gualdrón, D. A.; Wang, T. C.; García-Holley, P.; Sawelewa, R. M.; Argueta, E.; Snurr, R. Q.; Hupp, J. T.; Yildirim, T.; Farha, O. K. Understanding Volumetric and Gravimetric Hydrogen Adsorption Trade-off in Metal-Organic Frameworks. *ACS Appl. Mater. Interfaces* **2017**, *9*, 33419–33428. https://doi.org/10.1021/acsami.7b01190.

(50) Sun, D.; Ma, S.; Ke, Y.; Collins, D. J.; Zhou, H. C. An Interweaving MOF with High Hydrogen Uptake. *J. Am. Chem. Soc.* **2006**, *128*, 3896–3897. https://doi.org/10.1021/ja058777l.

(51) Ma, S.; Eckert, J.; Forster, P. M.; Ji, W. Y.; Young, K. H.; Chang, J. S.; Collier, C. D.; Parise, J. B.; Zhou, H. C. Further Investigation of the Effect of Framework Catenation on Hydrogen Uptake in Metal - Organic Frameworks. *J. Am. Chem. Soc.* **2008**, *130*, 15896–15902. https://doi.org/10.1021/ja803492q.

(52) Wang, X. Sen; Shengqian, M.; Rauch, K.; Simmons, J. M.; Yuan, D.; Wang, X.; Yildirim, T.; Cole, W. C.; López, J. J.; De Meijere, A.; Zhou, H. C. Metal-Organic Frameworks Based on Double-Bond-Coupled Di-Isophthalate Linkers with High Hydrogen and Methane Uptakes. *Chem. Mater.* **2008**, *20*, 3145–3152. https://doi.org/10.1021/cm800403d.

(53) Ma, S.; Simmons, J. M.; Sun, D.; Yuan, D.; Zhou, H. C. Porous Metal-Organic Frameworks Based on an Anthracene Derivative: Syntheses, Structure Analysis, and Hydrogen Sorption Studies. *Inorg. Chem.* **2009**, *48*, 5263–5268. https://doi.org/10.1021/ic900217t.

(54) Sun, D.; Ma, S.; Simmons, J. M.; Li, J. R.; Yuan, D.; Zhou, H. C. An Unusual Case of Symmetry-Preserving Isomerism. *Chem. Commun.* **2010**, *46*, 1329–1331. https://doi.org/10.1039/b920995f.

(55) Zhuang, W.; Ma, S.; Wang, X. Sen; Yuan, D.; Li, J. R.; Zhao, D.; Zhou, H. C. Introduction of Cavities up to 4 Nm into a Hierarchically-Assembled Metal-Organic Framework Using an Angular, Tetratopic Ligand. *Chem. Commun.* **2010**, *46*, 5223–5225. https://doi.org/10.1039/c0cc00779j.

(56) Zhao, D.; Yuan, D.; Yakovenko, A.; Zhou, H. C. A NbO-Type Metal-Organic Framework Derived from a Polyyne-Coupled Di-Isophthalate Linker Formed in Situ. *Chem. Commun.* **2010**, *46*, 4196–4198. https://doi.org/10.1039/c002767g.

(57) Yuan, D.; Zhao, D.; Sun, D.; Zhou, H.-C. An Isoreticular Series of Metal-Organic Frameworks with Dendritic Hexacarboxylate Ligands and Exceptionally High Gas-Uptake Capacity. *Angew. Chemie* **2010**, *122*, 5485–5489. https://doi.org/10.1002/ange.201001009.

(58) Zhang, P.; Li, B.; Zhao, Y.; Meng, X.; Zhang, T. A Novel (3,36)-Connected and Self-Interpenetrated Metal-Organic Framework with High Thermal Stability and Gas-Sorption Capabilities. *Chem. Commun.* **2011**, *47*, 7722–7724. https://doi.org/10.1039/c1cc11996f.

(59) Perles, J.; Iglesias, M.; Martín-Luengo, M. Á.; Monge, M. A.; Ruiz-Valero, C.; Snejko, N. Metal-Organic Scandium Framework: Useful Material for Hydrogen Storage and Catalysis. *Chem. Mater.* **2005**, *17*, 5837–5842. https://doi.org/10.1021/cm051362e.

(60) Lee, E. Y.; Jang, S. Y.; Suh, M. P. Multifunctionality and Crystal Dynamics of a Highly Stable, Porous Metal-Organic Framework [Zn4O(NTB)2]. *J. Am. Chem. Soc.* **2005**, *127*, 6374–6381. https://doi.org/10.1021/ja043756x.

(61) Lee, Y.-G.; Moon, H. R.; Cheon, Y. E.; Suh, M. P. A Comparison of the H 2 Sorption Capacities of Isostructural Metal-Organic Frameworks With and Without Accessible Metal Sites: [{Zn 2 (Abtc)(Dmf) 2 } 3] and [{Cu 2 (Abtc)(Dmf) 2 } 3] versus [{Cu 2 (Abtc)} 3] . *Angew. Chemie* **2008**, *120*, 7855–7859. https://doi.org/10.1002/ange.200801488.

(62) Park, H. J.; Cheon, Y. E.; Suh, M. P. Post-Synthetic Reversible Incorporation of Organic Linkers into Porous Metal-Organic Frameworks through Single-Crystal-to-Single-Crystal Transformations and Modification of Gas-Sorption Properties. *Chem. - A Eur. J.* **2010**, *16*, 11662–11669. https://doi.org/10.1002/chem.201001549.

(63) Kim, T. K.; Suh, M. P. Selective CO2 Adsorption in a Flexible Non-Interpenetrated Metal-Organic Framework. *Chem. Commun.* **2011**, *47*, 4258–4260. https://doi.org/10.1039/c0cc05199c.

(64) Prasad, T. K.; Hong, D. H.; Suh, M. P. High Gas Sorption and Metal-Ion Exchange of Microporous Metal-Organic Frameworks with Incorporated Imide Groups. *Chem. - A Eur. J.* **2010**, *16*, 14043–14050. https://doi.org/10.1002/chem.201002135.

(65) Ahmed, A.; Seth, S.; Purewal, J.; Wong-Foy, A. G.; Veenstra, M.; Matzger, A. J.; Siegel, D. J. Exceptional Hydrogen Storage Achieved by Screening Nearly Half a Million Metal-Organic Frameworks. *Nat. Commun.* **2019**, *10*. https://doi.org/10.1038/s41467-019-09365-w.

(66) Park, H. J.; Lim, D. W.; Yang, W. S.; Oh, T. R.; Suh, M. P. A Highly Porous Metal-Organic Framework: Structural Transformations of a Guest-Free MOF Depending on Activation Method and Temperature. *Chem. - A Eur. J.* **2011**, *17*, 7251–7260. https://doi.org/10.1002/chem.201003376.

(67) Kramer, M.; Schwarz, U.; Kaskel, S. Synthesis and Properties of the Metal-Organic Framework Mo 3(BTC)2 (TUDMOF-1). *J. Mater. Chem.* **2006**, *16*, 2245–2248. https://doi.org/10.1039/b601811d.

(68) Koh, K.; Wong-Foy, A. G.; Matzger, A. J. A Porous Coordination Copolymer with over 5000 m 2/g BET Surface Area. *J. Am. Chem. Soc.* **2009**, *131*, 4184–4185. https://doi.org/10.1021/ja809985t.

(69) Guo, Z.; Wu, H.; Srinivas, G.; Zhou, Y.; Xiang, S.; Chen, Z.; Yang, Y.; Zhou, W.; O'Keeffe, M.; Chen, B. A Metal-Organic Framework with Optimized Open Metal Sites and Pore Spaces for High Methane Storage at Room Temperature. *Angew. Chemie - Int. Ed.* **2011**, *50*, 3178–3181. https://doi.org/10.1002/anie.201007583.

(70) Wong-Foy, A. G.; Lebel, O.; Matzger, A. J. Porous Crystal Derived from a Tricarboxylate Linker with Two Distinct Binding Motifs. *J. Am. Chem. Soc.* **2007**, *129*, 15740–15741. https://doi.org/10.1021/ja0753952.

(71) Lou, J.; Xu, H.; Liu, Y.; Zhao, Y.; Daemen, L. L.; Brown, C.; Timofeeva, T. V.; Ma, S.; Zhou, H. C. Hydrogen Adsorption in a Highly Stable Porous Rare-Earth Metal-Organic Framework: Sorption Properties and Neutron Diffraction Studies. *J. Am. Chem. Soc.* **2008**, *130*, 9626–9627.

https://doi.org/10.1021/ja801411f.

(72) Zhou, W.; Wu, H.; Hartman, M. R.; Yildirim, T. Hydrogen and Methane Adsorption in Metal-Organic Frameworks: A High-Pressure Volumetric Study. *J. Phys. Chem. C* **2007**, *111*, 16131–16137. https://doi.org/10.1021/jp074889i.

(73) Panchariya, D. K.; Rai, R. K.; Anil Kumar, E.; Singh, S. K. Core-Shell Zeolitic Imidazolate Frameworks for Enhanced Hydrogen Storage. *ACS Omega* **2018**, *3*, 167–175. https://doi.org/10.1021/acsomega.7b01693.

(74) Chen, B.; Ma, S.; Zapata, F.; Lobkovsky, E. B.; Yang, J. Hydrogen Adsorption in an Interpenetrated Dynamic Metal-Organic Framework. *Inorg. Chem.* **2006**, *45*, 5718–5720. https://doi.org/10.1021/ic060437t.

(75) Liu, X.; Oh, M.; Lah, M. S. Size- and Shape-Selective Isostructural Microporous Metal-Organic Frameworks with Different Effective Aperture Sizes. *Inorg. Chem.* **2011**, *50*, 5044–5053. https://doi.org/10.1021/ic200328q.

(76) Chun, H.; Dybtsev, D. N.; Kim, H.; Kim, K. Synthesis, X-Ray Crystal Structures, and Gas Sorption Properties of Pillared Square Grid Nets Based on Paddle-Wheel Motifs: Implications for Hydrogen Storage in Porous Materials. *Chem. - A Eur. J.* **2005**, *11*, 3521–3529. https://doi.org/10.1002/chem.200401201.

(77) Takei, T.; Kawashima, J.; Ii, T.; Maeda, A.; Hasegawa, M.; Kitagawa, T.; Ohmura, T.; Ichikawa, M.; Hosoe, M.; Kanoya, I.; Mori, W. Hydrogen Adsorption Properties of Lantern-Type Dinuclear M(BDC)(DABCO) 1/2. *Bull. Chem. Soc. Jpn.* **2008**, *81*, 847–856. https://doi.org/10.1246/bcsj.81.847.

(78) Farha, O. K.; Mulfort, K. L.; Hupp, J. T. An Example of Node-Based Postassembly Elaboration of a Hydrogen-Sorbing, Metal- Organic Framework Material. *Inorg. Chem.* **2008**, *47*, 10223–10225.

(79) Lee, J.; Li, J.; Jagiello, J. Gas Sorption Properties of Microporous Metal Organic Frameworks. *J. Solid State Chem.* **2005**, *178*, 2527–2532. https://doi.org/10.1016/j.jssc.2005.07.002.

(80) Panda, T.; Pachfule, P.; Chen, Y.; Jiang, J.; Banerjee, R. Amino Functionalized Zeolitic Tetrazolate Framework (ZTF) with High Capacity for Storage of Carbon Dioxide. *Chem. Commun.* **2011**, *47*, 2011–2013. https://doi.org/10.1039/c0cc04169f.

(81) Debatin, F.; Thomas, A.; Kelling, A.; Hedin, N.; Bacsik, Z.; Senkovska, I.; Kaskel, S.; Junginger, M.; Müller, H.; Schilde, U.; Jäger, C.; Friedrich, A.; Holdt, H.-J. In Situ Synthesis of an Imidazolate-4-Amide-5-Imidate Ligand and Formation of a Microporous Zinc-Organic Framework with H 2 -and CO 2 -Storage Ability . *Angew. Chemie* **2010**, *122*, 1280–1284. https://doi.org/10.1002/ange.200906188.

(82) Alezi, D.; Belmabkhout, Y.; Suyetin, M.; Bhatt, P. M.; Weseliński, L. J.; Solovyeva, V.; Adil, K.; Spanopoulos, I.; Trikalitis, P. N.; Emwas, A. H.; Eddaoudi, M. MOF Crystal Chemistry Paving the Way to Gas Storage Needs: Aluminum-Based Soc -MOF for CH4, O2, and CO2 Storage. *J. Am. Chem. Soc.* **2015**, *137*, 13308–13318. https://doi.org/10.1021/jacs.5b07053.

(83) Sharma, M. K.; Senkovska, I.; Kaskel, S.; Bharadwaj, P. K. Three-Dimensional Porous Cd(II) Coordination Polymer with Large One-Dimensional Hexagonal Channels: High Pressure CH4 and H 2 Adsorption Studies. *Inorg. Chem.* **2011**, *50*, 539–544. https://doi.org/10.1021/ic101412p.

(84) Kondo, M.; Yoshitomi, T.; Matsuzaka, H.; Kitagawa, S.; Seki, K. Three-Dimensional Framework with Channeling Cavities for Small Molecules:$\{$[M2 (4, 4′-Bpy) 3 (NO3) 4]·xH2O$\}$ n (M□ Co, Ni, Zn). *Angew. Chemie Int. Ed. English* **1997**, *36*, 1725–1727.

(85) Kondo, M.; Shimamura, M.; Noro, S. I.; Minakoshi, S.; Asami, A.; Seki, K.; Kitagawa, S. Microporous Materials Constructed from the Interpenetrated Coordination Networks. Structures and Methane Adsorption Properties. *Chem. Mater.* **2000**, *12*, 1288–1299. https://doi.org/10.1021/cm990612m.

(86) Wang, H.; Getzschmann, J.; Senkovska, I.; Kaskel, S. Structural Transformation and High Pressure Methane Adsorption of Co2(1,4-Bdc)2dabco. *Microporous Mesoporous Mater.* **2008**, *116*, 653–657. https://doi.org/10.1016/j.micromeso.2008.05.037.

(87) Mason, J. A.; Veenstra, M.; Long, J. R. Evaluating Metal-Organic Frameworks for Natural Gas Storage. *Chem. Sci.* **2014**, *5*, 32–51. https://doi.org/10.1039/c3sc52633j.

(88) Seki, K.; Mori, W. Syntheses and Characterization of Microporous Coordination Polymers with Open Frameworks. *J. Phys. Chem. B* **2002**, *106*, 1380–1385. https://doi.org/10.1021/jp0130416.

(89) Seki, K. Design of an Adsorbent with an Ideal Pore Structure for Methane Adsorption Using Metal Complexes. *Chem. Commun.* **2001**, *1*, 1496–1497. https://doi.org/10.1039/b104204c.

(90) Senkovska, I.; Kaskel, S. High Pressure Methane Adsorption in the Metal-Organic Frameworks Cu3(Btc)2, Zn2(Bdc)2dabco, and Cr3F(H2O)2O(Bdc)3. *Microporous Mesoporous Mater.* **2008**, *112*, 108–115. https://doi.org/10.1016/j.micromeso.2007.09.016.

(91) Li, B.; Zhang, Z.; Li, Y.; Yao, K.; Zhu, Y.; Deng, Z.; Yang, F.; Zhou, X.; Li, G.; Wu, H.; Nijem, N.; Chabal, Y. J.; Lai, Z.; Han, Y.; Shi, Z.; Feng, S.; Li, J. Enhanced Binding Affinity, Remarkable Selectivity, and High Capacity of CO 2 by Dual Functionalization of a Rht-Type Metal-Organic Framework. *Angew. Chemie - Int. Ed.* **2012**, *51*, 1412–1415. https://doi.org/10.1002/anie.201105966.

(92) Senkovska, I.; Hoffmann, F.; Fröba, M.; Getzschmann, J.; Böhlmann, W.; Kaskel, S. New Highly Porous Aluminium Based Metal-Organic Frameworks: Al(OH)(Ndc) (Ndc = 2,6-Naphthalene Dicarboxylate) and Al(OH)(Bpdc) (Bpdc = 4,4′-Biphenyl Dicarboxylate). *Microporous Mesoporous Mater.* **2009**, *122*, 93–98. https://doi.org/10.1016/j.micromeso.2009.02.020.

(93) Klein, N.; Hoffmann, H. C.; Cadiau, A.; Getzschmann, J.; Lohe, M. R.; Paasch, S.; Heydenreich, T.; Adil, K.; Senkovska, I.; Brunner, E.; Kaskel, S. Structural Flexibility and Intrinsic Dynamics in the M2(2,6-Ndc) 2(Dabco) (M = Ni, Cu, Co, Zn) Metal-Organic Frameworks. *J. Mater. Chem.* **2012**, *22*, 10303–10312. https://doi.org/10.1039/c2jm15601f.

(94) Gedrich, K.; Senkovska, I.; Klein, N.; Stoeck, U.; Henschel, A.; Lohe, M. R.; Baburin, I. A.; Mueller, U.; Kaskel, S. A Highly Porous Metal-Organic Framework with Open Nickel Sites. *Angew. Chemie - Int. Ed.* **2010**, *49*, 8489–8492. https://doi.org/10.1002/anie.201001735.

(95) Grünker, R.; Senkovska, I.; Biedermann, R.; Klein, N.; Lohe, M. R.; Müller, P.; Kaskel, S. A Highly Porous Flexible Metal--Organic Framework with Corundum Topology. *Chem. Commun.* **2011**, *47*, 490–492.

(96) Klein, N.; Senkovska, I.; Baburin, I. A.; Grünker, R.; Stoeck, U.; Schlichtenmayer, M.; Streppel, B.; Mueller, U.; Leoni, S.; Hirscher, M.; Kaskel, S. Route to a Family of Robust, Non-Interpenetrated Metal-Organic Frameworks with Pto-like Topology. *Chem. - A Eur. J.* **2011**, *17*, 13007–13016. https://doi.org/10.1002/chem.201101383.

(97) Stoeck, U.; Krause, S.; Bon, V.; Senkovska, I.; Kaskel, S. A Highly Porous Metal–Organic Framework, Constructed from a Cuboctahedral Super-Molecular Building Block, with Exceptionally High Methane Uptake. *Chem. Commun.* **2012**, *48*, 10841–10843. https://doi.org/10.1039/c2cc34840c.

(98) Peng, Y.; Krungleviciute, V.; Eryazici, I.; Hupp, J. T.; Farha, O. K.; Yildirim, T. Methane Storage in Metal--Organic Frameworks: Current Records, Surprise Findings, and Challenges. *J. Am. Chem. Soc.* **2013**, *135*, 11887–11894.

(99) Eddaoudi, M.; Kim, J.; Rosi, N.; Vodak, D.; Wachter, J.; O'Keeffe, M.; Yaghi, O. M. Systematic Design of Pore Size and Functionality in Isoreticular MOFs and Their Application in Methane Storage. *Science (80-.).* **2002**, *295*, 469–472. https://doi.org/10.1126/science.1067208.

(100) Feldblyum, J. I.; Dutta, D.; Wong-Foy, A. G.; Dailly, A.; Imirzian, J.; Gidley, D. W.; Matzger, A. J. Interpenetration, Porosity, and High-Pressure Gas Adsorption in Zn 4O(2,6-Naphthalene Dicarboxylate)3. *Langmuir* **2013**, *29*, 8146–8153. https://doi.org/10.1021/la401323t.

(101) Bolinois, L.; Kundu, T.; Wang, X.; Wang, Y.; Hu, Z.; Koh, K.; Zhao, D. Breathing-Induced New Phase Transition in an MIL-53(Al)-NH2 Metal-Organic Framework under High Methane Pressures. *Chem. Commun.* **2017**, *53*, 8118–8121. https://doi.org/10.1039/c7cc02743e.

(102) Bourrelly, S.; Llewellyn, P. L.; Serre, C.; Millange, F.; Loiseau, T.; Férey, G. Different Adsorption Behaviors of Methane and Carbon Dioxide in the Isotypic Nanoporous Metal Terephthalates MIL-53 and MIL-47. *J. Am. Chem. Soc.* **2005**, *127*, 13519–13521. https://doi.org/10.1021/ja054668v.

(103) Llewellyn, P. L.; Bourrelly, S.; Serre, C.; Vimont, A.; Daturi, M.; Hamon, L.; De Weireld, G.; Chang, J.-S.; Hong, D.-Y.; Kyu Hwang, Y.; others. High Uptakes of CO2 and CH4 in Mesoporous Metal-Organic Frameworks MIL-100 and MIL-101. *Langmuir* **2008**, *24*, 7245–7250.

(104) Wiersum, A. D.; Chang, J. S.; Serre, C.; Llewellyn, P. L. An Adsorbent Performance Indicator as a First Step Evaluation of Novel Sorbents for Gas Separations: Application to Metal-Organic Frameworks. *Langmuir* **2013**, *29*, 3301–3309. https://doi.org/10.1021/la3044329.

(105) Wu, H.; Zhou, W.; Yildirim, T. High-Capacity Methane Storage in Metal-Organic Frameworks M2(Dhtp): The Important Role of Open Metal Sites. *J. Am. Chem. Soc.* **2009**, *131*, 4995–5000. https://doi.org/10.1021/ja900258t.

(106) He, Y.; Zhou, W.; Yildirim, T.; Chen, B. A Series of Metal--Organic Frameworks with High Methane Uptake and an Empirical Equation for Predicting Methane Storage Capacity. *Energy \& Environ. Sci.* **2013**, *6*, 2735–2744.

(107) Lu, Z.; Du, L.; Tang, K.; Bai, J. High H2 and CH4 Adsorption Capacity of a Highly Porous (2, 3, 4)-Connected Metal--Organic Framework. *Cryst. growth*

\& *Des.* **2013**, *13*, 2252–2255.

(108) He, Y.; Zhou, W.; Qian, G.; Chen, B. Methane Storage in Metal-Organic Frameworks. *Chem. Soc. Rev.* **2014**, *43*, 5657–5678. https://doi.org/10.1039/c4cs00032c.

(109) Peng, Y.; Srinivas, G.; Wilmer, C. E.; Eryazici, I.; Snurr, R. Q.; Farha, O. K.; Yildirim, T. Simultaneously High Gravimetric and Volumetric Methane Uptake Characteristics of the Metal-Organic Framework NU-111. *Chem. Commun.* **2013**, *49*, 2992–2994. https://doi.org/10.1039/c3cc40819a.

(110) Wilmer, C. E.; Farha, O. K.; Yildirim, T.; Eryazici, I.; Krungleviciute, V.; Sarjeant, A. A.; Snurr, R. Q.; Hupp, J. T. Gram-Scale, High-Yield Synthesis of a Robust Metal-Organic Framework for Storing Methane and Other Gases. *Energy Environ. Sci.* **2013**, *6*, 1158–1163. https://doi.org/10.1039/c3ee24506c.

(111) Barin, G.; Krungleviciute, V.; Gomez-Gualdron, D. A.; Sarjeant, A. A.; Snurr, R. Q.; Hupp, J. T.; Yildirim, T.; Farha, O. K. Isoreticular Series of (3,24)-Connected Metal-Organic Frameworks: Facile Synthesis and High Methane Uptake Properties. *Chem. Mater.* **2014**, *26*, 1912–1917. https://doi.org/10.1021/cm404155s.

(112) Gomez-Gualdron, D. A.; Gutov, O. V.; Krungleviciute, V.; Borah, B.; Mondloch, J. E.; Hupp, J. T.; Yildirim, T.; Farha, O. K.; Snurr, R. Q. Computational Design of Metal-Organic Frameworks Based on Stable Zirconium Building Units for Storage and Delivery of Methane. *Chem. Mater.* **2014**, *26*, 5632–5639. https://doi.org/10.1021/cm502304e.

(113) Lu, W.; Yuan, D.; Makal, T. A.; Li, J.-R.; Zhou, H.-C. A Highly Porous and Robust (3,3,4)-Connected Metal-Organic Framework Assembled with a 90° Bridging-Angle Embedded Octacarboxylate Ligand. *Angew. Chemie* **2012**, *124*, 1612–1616. https://doi.org/10.1002/ange.201106615.

(114) Prasad, T. K.; Suh, M. P. Control of Interpenetration and Gas-Sorption Properties of Metal-Organic Frameworks by a Simple Change in Ligand Design. *Chem. - A Eur. J.* **2012**, *18*, 8673–8680. https://doi.org/10.1002/chem.201200456.

(115) Das, M. C.; Xu, H.; Wang, Z.; Srinivas, G.; Zhou, W.; Yue, Y. F.; Nesterov, V. N.; Qian, G.; Chen, B. A Zn4O-Containing Doubly Interpenetrated Porous Metal-Organic Framework for Photocatalytic Decomposition of Methyl Orange. *Chem. Commun.* **2011**, *47*, 11715–11717. https://doi.org/10.1039/c1cc12802g.

(116) He, Y.; Xiang, S.; Zhang, Z.; Xiong, S.; Wu, C.; Zhou, W.; Yildirim, T.; Krishna, R.; Chen, B. A Microporous Metal-Organic Framework Assembled from an Aromatic Tetracarboxylate for H2 Purification. *J. Mater. Chem. A* **2013**, *1*, 2543–2551. https://doi.org/10.1039/c2ta01260j.

(117) Li, B.; Wen, H. M.; Wang, H.; Wu, H.; Yildirim, T.; Zhou, W.; Chen, B. Porous Metal-Organic Frameworks with Lewis Basic Nitrogen Sites for High-Capacity Methane Storage. *Energy Environ. Sci.* **2015**, *8*, 2504–2511. https://doi.org/10.1039/c5ee01531f.

(118) Wen, H. M.; Li, B.; Yuan, D.; Wang, H.; Yildirim, T.; Zhou, W.; Chen, B. A Porous Metal-Organic Framework with an Elongated Anthracene Derivative Exhibiting a High Working Capacity for the Storage of Methane. *J. Mater. Chem. A* **2014**, *2*, 11516–11522. https://doi.org/10.1039/c4ta01860e.

(119) Chang, G.; Li, B.; Wang, H.; Bao, Z.; Yildirim, T.; Yao, Z.; Xiang, S.; Zhou, W.; Chen, B. A Microporous Metal-Organic Framework with Polarized Trifluoromethyl Groups for High Methane Storage. *Chem. Commun.* **2015**, *51*, 14789–14792. https://doi.org/10.1039/c5cc05850c.

(120) Duan, X.; Wu, C.; Xiang, S.; Zhou, W.; Yildirim, T.; Cui, Y.; Yang, Y.; Chen, B.; Qian, G. Novel Microporous Metal-Organic Framework Exhibiting High Acetylene and Methane Storage Capacities. *Inorg. Chem.* **2015**, *54*, 4377–4381. https://doi.org/10.1021/acs.inorgchem.5b00194.

(121) Song, C.; Ling, Y.; Feng, Y.; Zhou, W.; Yildirim, T.; He, Y. A NbO-Type Metal--Organic Framework Exhibiting High Deliverable Capacity for Methane Storage. *Chem. Commun.* **2015**, *51*, 8508–8511.

(122) Park, M.; Moon, D.; Yoon, J. W.; Chang, J. S.; Lah, M. S. A Metal-Organic Framework Based on an Unprecedented Nonanuclear Cluster as a Secondary Building Unit: Structure and Gas Sorption Behavior. *Chem. Commun.* **2009**, 2026–2028. https://doi.org/10.1039/b818648k.

(123) An, J.; Rosi, N. L. Tuning MOF CO2 Adsorption Properties via Cation Exchange. *J. Am. Chem. Soc.* **2010**, *132*, 5578–5579. https://doi.org/10.1021/ja1012992.

(124) Si, X.; Jiao, C.; Li, F.; Zhang, J.; Wang, S.; Liu, S.; Li, Z.; Sun, L.; Xu, F.; Gabelica, Z.; Schick, C. High and Selective CO 2 Uptake, H 2 Storage and Methanol Sensing on the Amine-Decorated 12-Connected MOF CAU-1. *Energy Environ. Sci.* **2011**, *4*, 4522–4527. https://doi.org/10.1039/c1ee01380g.

(125) Wu, C. De; Lin, W. Highly Porous, Homochiral Metal-Organic Frameworks: Solvent-Exchange- Induced Single-Crystal to Single-Crystal Transformations. *Angew. Chemie - Int. Ed.* **2005**, *44*, 1958–1961. https://doi.org/10.1002/anie.200462711.

(126) Hou, L.; Shi, W. J.; Wang, Y. Y.; Guo, Y.; Jin, C.; Shi, Q. Z. A Rod Packing Microporous Metal-Organic Framework: Unprecedented Ukv Topology, High Sorption Selectivity and Affinity for CO2. *Chem. Commun.* **2011**, *47*, 5464–5466. https://doi.org/10.1039/c1cc10990a.

(127) Tian, J.; Motkuri, R. K.; Thallapally, P. K.; McGrail, B. P. Metal-Organic Framework Isomers with Diamondoid Networks Constructed of a Semirigid Tetrahedral Linker. *Cryst. Growth Des.* **2010**, *10*, 5327–5333. https://doi.org/10.1021/cg101222w.

(128) Pachfule, P.; Panda, T.; Dey, C.; Banerjee, R. Structural Diversity in a Series of Metal--Organic Frameworks (MOFs) Composed of Divalent Transition Metals, 4, 4′-Bipyridine and a Flexible Carboxylic Acid. *CrystEngComm* **2010**, *12*, 2381–2389.

(129) Tian, Y. Q.; Yao, S. Y.; Gu, D.; Cui, K. H.; Guo, D. W.; Zhang, G.; Chen, Z. X.; Zhao, D. Y. Cadmium Imidazolate Frameworks with Polymorphism, High Thermal Stability, and a Large Surface Area. *Chem. - A Eur. J.* **2010**, *16* (4), 1137–1141. https://doi.org/10.1002/chem.200902729.

(130) Herm, Z. R.; Swisher, J. A.; Smit, B.; Krishna, R.; Long, J. R. Metal-Organic Frameworks as Adsorbents for Hydrogen Purification and Precombustion Carbon Dioxide Capture. *J. Am. Chem. Soc.* **2011**, *133*, 5664–5667. https://doi.org/10.1021/ja111411q.

(131) Chen, S. S.; Chen, M.; Takamizawa, S.; Wang, P.; Lv, G. C.; Sun, W. Y. Porous Cobalt(II)-Imidazolate Supramolecular Isomeric Frameworks with Selective Gas Sorption Property. *Chem. Commun.* **2011**, *47*, 4902–4904. https://doi.org/10.1039/c1cc10899a.

(132) Caskey, S. R.; Wong-Foy, A. G.; Matzger, A. J. Dramatic Tuning of Carbon Dioxide Uptake via Metal Substitution in a Coordination Polymer with Cylindrical Pores. *J. Am. Chem. Soc.* **2008**, *130*, 10870–10871. https://doi.org/10.1021/ja8036096.

(133) Lin, Q.; Wu, T.; Zheng, S. T.; Bu, X.; Feng, P. Single-Walled Polytetrazolate Metal-Organic Channels with High Density of Open Nitrogen-Donor Sites and Gas Uptake. *J. Am. Chem. Soc.* **2012**, *134*, 784–787. https://doi.org/10.1021/ja2092882.

(134) García-Ricard, O. J.; Hernández-Maldonado, A. J. Temperature , Textural Properties , and CO 2 Adsorption at Low Pressure Range. *J. Phys. Chem. C* **2010**, *114*, 1827–1834.

(135) Zhao, X.; Bu, X.; Zhai, Q. G.; Tran, H.; Feng, P. Pore Space Partition by Symmetry-Matching Regulated Ligand Insertion and Dramatic Tuning on Carbon Dioxide Uptake. *J. Am. Chem. Soc.* **2015**, *137*, 1396–1399. https://doi.org/10.1021/ja512137t.

(136) Zheng, S. T.; Bu, J. T.; Li, Y.; Wu, T.; Zuo, F.; Feng, P.; Bu, X. Pore Space Partition and Charge Separation in Cage-within-Cage Indium-Organic Frameworks with High CO2 Uptake. *J. Am. Chem. Soc.* **2010**, *132*, 17062–17064. https://doi.org/10.1021/ja106903p.

(137) Navarro, J. A. R.; Barea, E.; Salas, J. M.; Masciocchi, N.; Galli, S.; Sironi, A.; Ania, C. O.; Parra, J. B. H2, N2, CO, and CO2 Sorption Properties of a Series of Robust Sodalite-Type Microporous Coordination Polymers. *Inorg. Chem.* **2006**, *45*, 2397–2399. https://doi.org/10.1021/ic060049r.

(138) Xiang, S.; He, Y.; Zhang, Z.; Wu, H.; Zhou, W.; Krishna, R.; Chen, B. Microporous Metal-Organic Framework with Potential for Carbon Dioxide Capture at Ambient Conditions. *Nat. Commun.* **2012**, *3*, 1–9.

(139) Kitaura, R.; Matsuda, R.; Kubota, Y.; Kitagawa, S.; Takata, M.; Kobayashi, T. C.; Suzuki, M. Formation and Characterization of Crystalline Molecular Arrays of Gas Molecules in a 1-Dimensional Ultramicropore of a Porous Copper Coordination Polymer. *J. Phys. Chem. B* **2005**, *109*, 23378–23385. https://doi.org/10.1021/jp054411j.

(140) Zheng, B.; Yang, Z.; Bai, J.; Li, Y.; Li, S. High and Selective Co2 Capture by Two Mesoporous Acylamide-Functionalized Rht-Type Metal-Organic Frameworks. *Chem. Commun.* **2012**, *48*, 7025–7027. https://doi.org/10.1039/c2cc17593b.

(141) Lincke, J.; Lässig, D.; Möllmer, J.; Reichenbach, C.; Puls, A.; Möller, A.; Gläser, R.; Kalies, G.; Staudt, R.; Krautscheid, H. A Novel Copper-Based MOF Material: Synthesis, Characterization and Adsorption Studies. *Microporous mesoporous Mater.* **2011**, *142*, 62–69.

(142) Demessence, A.; D'Alessandro, D. M.; Foo, M. L.; Long, J. R. Strong CO2 Binding in a Water-Stable, Triazolate-Bridged Metal-Organic Framework Functionalized with Ethylenediamine. *J. Am. Chem. Soc.* **2009**, *131*, 8784–8786. https://doi.org/10.1021/ja903411w.

(143) Zheng, B.; Bai, J.; Duan, J.; Wojtas, L.; Zaworotko, M. J. Enhanced CO2 Binding Affinity of a High-Uptake Rht-Type Metal- Organic Framework Decorated with Acylamide Groups. *J. Am. Chem. Soc.* **2011**, *133*, 748–751.

(144) Sumida, K.; Horike, S.; Kaye, S. S.; Herm, Z. R.; Queen, W. L.; Brown, C. M.; Grandjean, F.; Long, G. J.; Dailly, A.; Long, J. R. Hydrogen Storage and

Carbon Dioxide Capture in an Iron-Based Sodalite-Type Metal-Organic Framework (Fe-BTT) Discovered via High-Throughput Methods. *Chem. Sci.* **2010**, *1*, 184–191. https://doi.org/10.1039/c0sc00179a.

(145) Mason, J. A.; McDonald, T. M.; Bae, T. H.; Bachman, J. E.; Sumida, K.; Dutton, J. J.; Kaye, S. S.; Long, J. R. Application of a High-Throughput Analyzer in Evaluating Solid Adsorbents for Post-Combustion Carbon Capture via Multicomponent Adsorption of CO2, N2, and H2O. *J. Am. Chem. Soc.* **2015**, *137*, 4787–4803. https://doi.org/10.1021/jacs.5b00838.

(146) Sumida, K.; Rogow, D. L.; Mason, J. A.; McDonald, T. M.; Bloch, E. D.; Herm, Z. R.; Bae, T.-H.; Long, J. R. Carbon Dioxide Capture in Metal--Organic Frameworks. *Chem. Rev.* **2012**, *112*, 724–781.

(147) Liang, L.; Liu, C.; Jiang, F.; Chen, Q.; Zhang, L.; Xue, H.; Jiang, H.-L.; Qian, J.; Yuan, D.; Hong, M. Carbon Dioxide Capture and Conversion by an Acid-Base Resistant Metal-Organic Framework. *Nat. Commun.* **2017**, *8*, 1–10.

(148) Fernandez, C. A.; Thallapally, P. K.; Motkuri, R. K.; Nune, S. K.; Sumrak, J. C.; Tian, J.; Liu, J. Gas-Induced Expansion and Contraction of a Fluorinated Metal-Organic Framework. *Cryst. Growth Des.* **2010**, *10*, 1037–1039. https://doi.org/10.1021/cg9014948.

(149) Millward, A. R.; Yaghi, O. M. Metal-Organic Frameworks with Exceptionally High Capacity for Storage of Carbon Dioxide at Room Temperature. *J. Am. Chem. Soc.* **2005**, *127*, 17998–17999. https://doi.org/10.1021/ja0570032.

(150) Ling, Y.; Deng, M.; Chen, Z.; Xia, B.; Liu, X.; Yang, Y.; Zhou, Y.; Weng, L. Enhancing Co2 Adsorption of a Zn-Phosphonocarboxylate Framework by Pore Space Partitions. *Chem. Commun.* **2013**, *49*, 78–80. https://doi.org/10.1039/c2cc37174j.

(151) Qin, J. S.; Du, D. Y.; Li, W. L.; Zhang, J. P.; Li, S. L.; Su, Z. M.; Wang, X. L.; Xu, Q.; Shao, K. Z.; Lan, Y. Q. N-Rich Zeolite-like Metal-Organic Framework with Sodalite Topology: High CO2 Uptake, Selective Gas Adsorption and Efficient Drug Delivery. *Chem. Sci.* **2012**, *3*, 2114–2118. https://doi.org/10.1039/c2sc00017b.

(152) Tan, Y. X.; Wang, F.; Kang, Y.; Zhang, J. Dynamic Microporous Indium(III)-4,4'-Oxybis(Benzoate) Framework with High Selectivity for the Adsorption of CO2 over N2. *Chem. Commun.* **2011**, *47*, 770–772. https://doi.org/10.1039/c0cc04095a.

(153) Möllmer, J.; Celer, E. B.; Luebke, R.; Cairns, A. J.; Staudt, R.; Eddaoudi, M.; Thommes, M. Insights on Adsorption Characterization of Metal-Organic Frameworks: A Benchmark Study on the Novel Soc-MOF. *Microporous Mesoporous Mater.* **2010**, *129*, 345–353.

(154) Chen, S.; Zhang, J.; Wu, T.; Feng, P.; Bu, X. Multiroute Synthesis of Porous Anionic Frameworks and Size-Tunable Extraframework Organic Cation-Controlled Gas Sorption Properties. *J. Am. Chem. Soc.* **2009**, *131*, 16027–16029. https://doi.org/10.1021/ja906302t.

(155) Orefuwa, S.; Iriowen, E.; Yang, H.; Wakefield, B.; Goudy, A. Effects of Nitro-Functionalization on the Gas Adsorption Properties of Isoreticular Metal-Organic Framework-Eight (IRMOF-8). *Microporous Mesoporous Mater.* **2013**, *177*, 82–90. https://doi.org/10.1016/j.micromeso.2013.04.023.

(156) Bae, Y. S.; Dubbeldam, D.; Nelson, A.; Walton, K. S.; Hupp, J. T.; Snurr, R. Q. Strategies for Characterization of Large-Pore Metal-Organic Frameworks by Combined Experimental and Computational Methods. *Chem. Mater.* **2009**, *21*, 4768–4777. https://doi.org/10.1021/cm803218f.

(157) Fracaroli, A. M.; Furukawa, H.; Suzuki, M.; Dodd, M.; Okajima, S.; Gándara, F.; Reimer, J. A.; Yaghi, O. M. Metal-Organic Frameworks with Precisely Designed Interior for Carbon Dioxide Capture in the Presence of Water. *J. Am. Chem. Soc.* **2014**, *136*, 8863–8866.

(158) Chen, C. X.; Zheng, S. P.; Wei, Z. W.; Cao, C. C.; Wang, H. P.; Wang, D.; Jiang, J. J.; Fenske, D.; Su, C. Y. A Robust Metal–Organic Framework Combining Open Metal Sites and Polar Groups for Methane Purification and CO2/Fluorocarbon Capture. *Chem. - A Eur. J.* **2017**, *23*, 4060–4064. https://doi.org/10.1002/chem.201606038.

(159) Chen, C.-X.; Wei, Z.; Jiang, J.-J.; Fan, Y.-Z.; Zheng, S.-P.; Cao, C.-C.; Li, Y.-H.; Fenske, D.; Su, C.-Y. Precise Modulation of the Breathing Behavior and Pore Surface in Zr-MOFs by Reversible Post-Synthetic Variable-Spacer Installation to Fine-Tune the Expansion Magnitude and Sorption Properties. *Angew. Chemie* **2016**, *128*, 10086–10090.

(160) Zhang, J. P.; Chen, X. M. Optimized Acetylene/Carbon Dioxide Sorption in a Dynamic Porous Crystal. *J. Am. Chem. Soc.* **2009**, *131*, 5516–5521. https://doi.org/10.1021/ja8089872.

(161) Liao, P. Q.; Zhou, D. D.; Zhu, A. X.; Jiang, L.; Lin, R. B.; Zhang, J. P.; Chen, X. M. Strong and Dynamic CO 2 Sorption in a Flexible Porous Framework Possessing Guest Chelating Claws. *J. Am. Chem. Soc.* **2012**, *134*, 17380–17383. https://doi.org/10.1021/ja3073512.

(162) Lin, J.-B.; Zhang, J.-P.; Chen, X.-M. Nonclassical Active Site for Enhanced Gas Sorption in Porous Coordination Polymer. *J. Am. Chem. Soc.* **2010**, *132*, 6654–6656.

(163) Lin, R. B.; Chen, D.; Lin, Y. Y.; Zhang, J. P.; Chen, X. M. A Zeolite-like Zinc Triazolate Framework with High Gas Adsorption and Separation Performance. *Inorg. Chem.* **2012**, *51*, 9950–9955. https://doi.org/10.1021/ic301463z.

(164) Cheon, Y. E.; Park, J.; Suh, M. P. Selective Gas Adsorption in a Magnesium-Based Metal-Organic Framework. *Chem. Commun.* **2009**, 5436–5438. https://doi.org/10.1039/b910228k.

(165) McDonald, T. M.; Lee, W. R.; Mason, J. A.; Wiers, B. M.; Hong, C. S.; Long, J. R. Capture of Carbon Dioxide from Air and Flue Gas in the Alkylamine-Appended Metal-Organic Framework Mmen-Mg 2(Dobpdc). *J. Am. Chem. Soc.* **2012**, *134*, 7056–7065. https://doi.org/10.1021/ja300034j.

(166) Mallick, A.; Saha, S.; Pachfule, P.; Roy, S.; Banerjee, R. Selective CO2 and H2 Adsorption in a Chiral Magnesium-Based Metal Organic Framework (Mg-MOF) with Open Metal Sites. *J. Mater. Chem.* **2010**, *20*, 9073–9080. https://doi.org/10.1039/c0jm01125h.

(167) Bao, Z.; Yu, L.; Ren, Q.; Lu, X.; Deng, S. Adsorption of CO2 and CH4 on a Magnesium-Based Metal Organic Framework. *J. Colloid Interface Sci.* **2011**, *353*, 549–556. https://doi.org/10.1016/j.jcis.2010.09.065.

(168) Dietzel, P. D. C.; Besikiotis, V.; Blom, R. Application of Metal-Organic Frameworks with Coordinatively Unsaturated Metal Sites in Storage and Separation of Methane and Carbon Dioxide. *J. Mater. Chem.* **2009**, *19*, 7362–7370. https://doi.org/10.1039/b911242a.

(169) Rallapalli, P.; Prasanth, K. P.; Patil, D.; Somani, R. S.; Jasra, R. V.; Bajaj, H. C. Sorption Studies of CO2, CH4, N2, CO, O2 and Ar on Nanoporous Aluminum Terephthalate [MIL-53(Al)]. *J. Porous Mater.* **2011**, *18*, 205–210. https://doi.org/10.1007/s10934-010-9371-7.

(170) Loiseau, T.; Lecroq, L.; Volkringer, C.; Marrot, J.; Férey, G.; Haouas, M.; Taulelle, F.; Bourrelly, S.; Llewellyn, P. L.; Latroche, M. MIL-96, a Porous Aluminum Trimesate 3D Structure Constructed from a Hexagonal Network of 18-Membered Rings and M3-Oxo-Centered Trinuclear Units. *J. Am. Chem. Soc.* **2006**, *128*, 10223–10230. https://doi.org/10.1021/ja0621086.

(171) Chowdhury, P.; Mekala, S.; Dreisbach, F.; Gumma, S. Adsorption of CO, CO2 and CH4 on Cu-BTC and MIL-101 Metal Organic Frameworks: Effect of Open Metal Sites and Adsorbate Polarity. *Microporous Mesoporous Mater.* **2012**, *152*, 246–252.

(172) Surblé, S.; Millange, F.; Serre, C.; Düren, T.; Latroche, M.; Bourrelly, S.; Llewellyn, P. L.; Férey, G. Synthesis of MIL-102, a Chromium Carboxylate Metal-Organic Framework, with Gas Sorption Analysis. *J. Am. Chem. Soc.* **2006**, *128*, 14889–14896. https://doi.org/10.1021/ja064343u.

(173) Volkringer, C.; Loiseau, T.; Haouas, M.; Taulelle, F.; Popov, D.; Burghammer, M.; Riekel, C.; Zlotea, C.; Cuevas, F.; Latroche, M.; Phanon, D.; Knöfelv, C.; Llewellyn, P. L.; Férey, G. Occurrence of Uncommon Infinite Chains Consisting of Edge-Sharing Octahedra in a Porous Metal Organic Framework-Type Aluminum Pyromellitate Al 4(OH)8[C10O8H2](MIL-120): Synthesis, Structure, and Gas Sorption Properties. *Chem. Mater.* **2009**, *21*, 5783–5791. https://doi.org/10.1021/cm9023106.

(174) McDonald, T. M.; D'Alessandro, D. M.; Krishna, R.; Long, J. R. Enhanced Carbon Dioxide Capture upon Incorporation of N,N'-Dimethylethylenediamine in the Metal-Organic Framework CuBTTri. *Chem. Sci.* **2011**, *2*, 2022–2028. https://doi.org/10.1039/c1sc00354b.

(175) Foo, M. L.; Matsuda, R.; Hijikata, Y.; Krishna, R.; Sato, H.; Horike, S.; Hori, A.; Duan, J.; Sato, Y.; Kubota, Y.; Takata, M.; Kitagawa, S. An Adsorbate Discriminatory Gate Effect in a Flexible Porous Coordination Polymer for Selective Adsorption of CO2 over C2H2. *J. Am. Chem. Soc.* **2016**, *138*, 3022–3030. https://doi.org/10.1021/jacs.5b10491.

(176) Hoi, R. M.; Kobayashi, N.; Myunghyun, P. S. Porous Metal-Organic Framework with Coordinatively Unsaturated Mn II Sites:Sorption Properties for Various Gases. *Inorg. Chem.* **2006**, *45*, 8672–8676. https://doi.org/10.1021/ic0611948.

(177) Bloch, E. D.; Britt, D.; Lee, C.; Doonan, C. J.; Uribe-Romo, F. J.; Furukawa, H.; Long, J. R.; Yaghi, O. M. Metal Insertion in a Microporous Metal-Organic Framework Lined with 2,2'-Bipyridine. *J. Am. Chem. Soc.* **2010**, *132*, 14382–14384. https://doi.org/10.1021/ja106935d.

(178) Lee, C. H.; Huang, H. Y.; Lee, J. J.; Huang, C. Y.; Kao, Y. C.; Lee, G. H.; Peng, S. M.; Jiang, J. C.; Chao, I.; Lu, K. L. Amide-CO2 Interaction Induced Gate-Opening Behavior for CO2 Adsorption in 2-Fold Interpenetrating Framework. *ChemistrySelect* **2016**, *1*, 2923–2929. https://doi.org/10.1002/slct.201600345.

(179) Saha, D.; Bao, Z.; Jia, F.; Deng, S. Adsorption of CO2, CH4, N2O, and N2 on MOF-5, MOF-177, and Zeolite 5A. *Environ. Sci. \& Technol.* **2010**, *44*, 1820–1826.

(180) Barcia, P. S.; Bastin, L.; Hurtado, E. J.; Silva, J. A. C.; Rodrigues, A. E.; Chen, B. Single and Multicomponent Sorption of CO2, CH4 and N2 in a Microporous Metal-Organic Framework. *Sep. Sci. Technol.* **2008**, *43*, 3494–3521. https://doi.org/10.1080/01496390802282347.

(181) Couck, S.; Denayer, J. F. M.; Baron, G. V.; Rémy, T.; Gascon, J.; Kapteijn, F. An Amine-Functionalized MIL-53 Metal-Organic Framework with Large Separation Power for CO2 and CH4. *J. Am. Chem. Soc.* **2009**, *131*, 6326–6327. https://doi.org/10.1021/ja900555r.

(182) Wu, C. De; Lin, W. Homochiral Porous Solids Based on 1D Coordination Polymers Built from 46-Membered Macrocycles. *Dalt. Trans.* **2006**, 4563–4569. https://doi.org/10.1039/b606817k.

(183) Chen, Z.; Liu, X.; Zhang, C.; Zhang, Z.; Liang, F. Structure, Adsorption and Magnetic Properties of Chiral Metal-Organic Frameworks Bearing Linear Trinuclear Secondary Building Blocks. *Dalt. Trans.* **2011**, *40*, 1911–1918. https://doi.org/10.1039/c0dt01278e.

(184) Nandi, S.; De Luna, P.; Daff, T. D.; Rother, J.; Liu, M.; Buchanan, W.; Hawari, A. I.; Woo, T. K.; Vaidhyanathan, R. A Single-Ligand Ultra-Microporous MOF for Precombustion CO2 Capture and Hydrogen Purification. *Sci. Adv.* **2015**, *1*, 1–10. https://doi.org/10.1126/sciadv.1500421.

(185) Duan, J.; Yang, Z.; Bai, J.; Zheng, B.; Li, Y.; Li, S. Highly Selective CO2 Capture of an Agw-Type Metal-Organic Framework with Inserted Amides: Experimental and Theoretical Studies. *Chem. Commun.* **2012**, *48*, 3058–3060. https://doi.org/10.1039/c2cc16231h.

(186) Alsmail, N. H.; Suyetin, M.; Yan, Y.; Cabot, R.; Krap, C. P.; Lü, J.; Easun, T. L.; Bichoutskaia, E.; Lewis, W.; Blake, A. J.; Schröder, M. Analysis of High and Selective Uptake of CO2 in an Oxamide-Containing {Cu2(OOCR)4}-Based Metal-Organic Framework. *Chem. - A Eur. J.* **2014**, *20*, 7317–7324. https://doi.org/10.1002/chem.201304005.

(187) Yang, S.; Lin, X.; Lewis, W.; Suyetin, M.; Bichoutskaia, E.; Parker, J. E.; Tang, C. C.; Allan, D. R.; Rizkallah, P. J.; Hubberstey, P.; Champness, N. R.; Mark Thomas, K.; Blake, A. J.; Schröder, M. A Partially Interpenetrated Metal-Organic Framework for Selective Hysteretic Sorption of Carbon Dioxide. *Nat. Mater.* **2012**, *11*, 710–716. https://doi.org/10.1038/nmat3343.

(188) Li, J. R.; Yu, J.; Lu, W.; Sun, L. B.; Sculley, J.; Balbuena, P. B.; Zhou, H. C. Porous Materials with Pre-Designed Single-Molecule Traps for CO 2 Selective Adsorption. *Nat. Commun.* **2013**, *4*, 1–8. https://doi.org/10.1038/ncomms2552.

(189) Park, J.; Yuan, D.; Pham, K. T.; Li, J. R.; Yakovenko, A.; Zhou, H. C. Reversible Alteration of CO 2 Adsorption upon Photochemical or Thermal Treatment in a Metal-Organic Framework. *J. Am. Chem. Soc.* **2012**, *134*, 99–102. https://doi.org/10.1021/ja209197f.

(190) Lv, D.; Shi, R.; Chen, Y.; Chen, Y.; Wu, H.; Zhou, X.; Xi, H.; Li, Z.; Xia, Q. Selective Adsorptive Separation of CO2/CH4 and CO2/N2 by a Water Resistant Zirconium-Porphyrin Metal-Organic Framework. *Ind. Eng. Chem. Res.* **2018**, *57*, 12215–12224. https://doi.org/10.1021/acs.iecr.8b02596.

(191) Navarro, J. A. R.; Barea, E.; Salas, J. M.; Masciocchi, N.; Galli, S.; Sironi, A.; Ania, C. O.; Parra, J. B. Borderline Microporous-Ultramicroporous Palladium(Ii) Coordination Polymer Networks. Effect of Pore Functionalisation on Gas Adsorption Properties. *J. Mater. Chem.* **2007**, *17*, 1939–1946. https://doi.org/10.1039/b617698d.

(192) Miller, S. R.; Wright, P. A.; Devic, T.; Serre, C.; Férey, G.; Llewellyn, P. L.; Denoyel, R.; Gaberova, L.; Filinchuk, Y. Single Crystal X-Ray Diffraction Studies of Carbon Dioxide and Fuel-Related Gases Adsorbed on the Small Pore Scandium Terephthalate Metal Organic Framework, Sc2 (O2CC6H4CO2) 3. *Langmuir* **2009**, *25*, 3618–3626.

(193) Carrington, E. J.; McAnally, C. A.; Fletcher, A. J.; Thompson, S. P.; Warren, M.; Brammer, L. Solvent-Switchable Continuous-Breathing Behaviour in a Diamondoid Metal-Organic Framework and Its Influence on CO2 versus CH4 Selectivity. *Nat. Chem.* **2017**, *9*, 882–889. https://doi.org/10.1038/nchem.2747.

(194) Suh, M. P.; Cheon, Y. E.; Lee, E. Y. Reversible Transformation of ZnII Coordination Geometry in a Single Crystal of Porous Metal-Organic Framework [Zn3(Ntb) 2(EtOH)2]·4EtOH. *Chem. - A Eur. J.* **2007**, *13*, 4208–4215. https://doi.org/10.1002/chem.200601534.

(195) Park, H. J.; Suh, M. P. Mixed-Ligand Metal-Organic Frameworks with Large Pores: Gas Sorption Properties and Single-Crystal-to-Single-Crystal Transformation on Guest Exchange. *Chem. - A Eur. J.* **2008**, *14*, 8812–8821. https://doi.org/10.1002/chem.200801064.

(196) Park, H. J.; Suh, M. P. Stepwise and Hysteretic Sorption of N2, O2, CO 2, and H2 Gases in a Porous Metal-Organic Framework [Zn2(BPnDC)2(Bpy)]. *Chem. Commun.* **2010**, *46*, 610–612. https://doi.org/10.1039/b913067e.

(197) Cheon, Y. E.; Suh, M. P. Selective Gas Adsorption in a Microporous Metal-Organic Framework Constructed of CoII4 Clusters. *Chem. Commun.* **2009**, 2296–2298. https://doi.org/10.1039/b900085b.

(198) Choi, H. S.; Suh, M. P. Highly Selective CO2 Capture in Flexible 3d Coordination Polymer Networks. *Angew. Chemie - Int. Ed.* **2009**, *48*, 6865–6869. https://doi.org/10.1002/anie.200902836.

(199) Yao, Q.; Su, J.; Cheung, O.; Liu, Q.; Hedin, N.; Zou, X. Interpenetrated Metal-Organic Frameworks and Their Uptake of CO2 at Relatively Low Pressures. *J. Mater. Chem.* **2012**, *22*, 10345–10351. https://doi.org/10.1039/c2jm15933c.

(200) Zhang, G.; Wei, G.; Liu, Z.; Oliver, S. R. J.; Fei, H. A Robust Sulfonate-Based Metal-Organic Framework with Permanent Porosity for Efficient CO2 Capture and Conversion. *Chem. Mater.* **2016**, *28*, 6276–6281. https://doi.org/10.1021/acs.chemmater.6b02511.

(201) Safarifard, V.; Rodríguez-Hermida, S.; Guillerm, V.; Imaz, I.; Bigdeli, M.; Tehrani, A. A.; Juanhuix, J.; Morsali, A.; Casco, M. E.; Silvestre-Albero, J.; Ramos-Fernandez, E. V.; Maspoch, D. Influence of the Amide Groups in the CO2/N2 Selectivity of a Series of Isoreticular, Interpenetrated Metal-Organic Frameworks. *Cryst. Growth Des.* **2016**, *16*, 6016–6023. https://doi.org/10.1021/acs.cgd.6b01054.

(202) Li, L.; Tang, S.; Wang, C.; Lv, X.; Jiang, M.; Wu, H.; Zhao, X. High Gas Storage Capacities and Stepwise Adsorption in a UiO Type Metal–Organic

Framework Incorporating Lewis Basic Bipyridyl Sites. *Chem. Commun.* **2014**, *50*, 2304–2307. https://doi.org/10.1039/c3cc48275h.

(203) Yazaydin, A. Ö.; Snurr, R. Q.; Park, T. H.; Koh, K.; Liu, J.; LeVan, M. D.; Benin, A. I.; Jakubczak, P.; Lanuza, M.; Galloway, D. B.; Low, J. J.; Willis, R. R. Screening of Metal-Organic Frameworks for Carbon Dioxide Capture from Flue Gas Using a Combined Experimental and Modeling Approach. *J. Am. Chem. Soc.* **2009**, *131*, 18198–18199. https://doi.org/10.1021/ja9057234.

(204) Mu, B.; Schoenecker, P. M.; Walton, K. S. Gas Adsorption Study on Mesoporous Metal- Organic Framework UMCM-1. *J. Phys. Chem. C* **2010**, *114*, 6464–6471.

(205) Arstad, B.; Fjellvåg, H.; Kongshaug, K. O.; Swang, O.; Blom, R. Amine Functionalised Metal Organic Frameworks (MOFs) as Adsorbents for Carbon Dioxide. *Adsorption* **2008**, *14*, 755–762. https://doi.org/10.1007/s10450-008-9137-6.

(206) Jiang, Z. R.; Wang, H.; Hu, Y.; Lu, J.; Jiang, H. L. Polar Group and Defect Engineering in a Metal-Organic Framework: Synergistic Promotion of Carbon Dioxide Sorption and Conversion. *ChemSusChem* **2015**, *8*, 878–885. https://doi.org/10.1002/cssc.201403230.

(207) Chen, Z.; Xiang, S.; Arman, H. D.; Mondal, J. U.; Li, P.; Zhao, D.; Chen, B. Three-Dimensional Pillar-Layered Copper(II) Metal-Organic Framework with Immobilized Functional OH Groups on Pore Surfaces for Highly Selective CO 2/CH4 and C2H2/CH4 Gas Sorption at Room Temperature. *Inorg. Chem.* **2011**, *50*, 3442–3446. https://doi.org/10.1021/ic102347b.

(208) Chen, Z.; Xiang, S.; Arman, H. D.; Li, P.; Zhao, D.; Chen, B. Significantly Enhanced CO2/CH4 Separation Selectivity within a 3D Prototype Metal-Organic Framework Functionalized with OH Groups on Pore Surfaces at Room Temperature. *Eur. J. Inorg. Chem.* **2011**, 2227–2231. https://doi.org/10.1002/ejic.201100034.

(209) He, Y.; Zhang, Z.; Xiang, S.; Fronczek, F. R.; Krishna, R.; Chen, B. A Microporous Metal-Organic Framework for Highly Selective Separation of Acetylene, Ethylene, and Ethane from Methane at Room Temperature. *Chem. - A Eur. J.* **2012**, *18*, 613–619. https://doi.org/10.1002/chem.201102734.

(210) Mulfort, K. L.; Farha, O. K.; Malliakas, C. D.; Kanatzidis, M. G.; Hupp, J. T. An Interpenetrated Framework Material with Hysteretic CO2 Uptake. *Chem. - A Eur. J.* **2010**, *16*, 276–281. https://doi.org/10.1002/chem.200902104.

(211) Banerjee, R.; Furukawa, H.; Britt, D.; Knobler, C.; O'Keeffe, M.; Yaghi, O. M. Control of Pore Size and Functionality in Isoreticular Zeolitic Imidazolate Frameworks and Their Carbon Dioxide Selective Capture Properties. *J. Am. Chem. Soc.* **2009**, *131*, 3875–3877. https://doi.org/10.1021/ja809459e.

(212) Morris, W.; Leung, B.; Furukawa, H.; Yaghi, O. K.; He, N.; Hayashi, H.; Houndonougbo, Y.; Asta, M.; Laird, B. B.; Yaghi, O. M. A Combined Experimental-Computational Investigation of Carbon Dioxide Capture in a Series of Isoreticular Zeolitic Imidazolate Frameworks. *J. Am. Chem. Soc.* **2010**, *132*, 11006–11008. https://doi.org/10.1021/ja104035j.

(213) Song, C.; He, Y.; Li, B.; Ling, Y.; Wang, H.; Feng, Y.; Krishna, R.; Chen, B. Enhanced CO2 Sorption and Selectivity by Functionalization of a NbO-Type

Metal-Organic Framework with Polarized Benzothiadiazole Moieties. *Chem. Commun.* **2014**, *50*, 12105–12108. https://doi.org/10.1039/c4cc05833j.

(214) Song, C.; Hu, J.; Ling, Y.; Feng, Y.; Krishna, R.; Chen, D.; He, Y. The Accessibility of Nitrogen Sites Makes a Difference in Selective CO 2 Adsorption of a Family of Isostructural Metal--Organic Frameworks. *J. Mater. Chem. A* **2015**, *3*, 19417–19426.

(215) Zhao, Y.; Wu, H.; Emge, T. J.; Gong, Q.; Nijem, N.; Chabal, Y. J.; Kong, L.; Langreth, D. C.; Liu, H.; Zeng, H.; Li, J. Enhancing Gas Adsorption and Separation Capacity through Ligand Functionalization of Microporous Metal-Organic Framework Structures. *Chem. - A Eur. J.* **2011**, *17*, 5101–5109. https://doi.org/10.1002/chem.201002818.

(216) Mosca, N.; Vismara, R.; Fernandes, J. A.; Tuci, G.; Di Nicola, C.; Domasevitch, K. V.; Giacobbe, C.; Giambastiani, G.; Pettinari, C.; Aragones-Anglada, M.; Moghadam, P. Z.; Fairen-Jimenez, D.; Rossin, A.; Galli, S. Nitro-Functionalized Bis(Pyrazolate) Metal–Organic Frameworks as Carbon Dioxide Capture Materials under Ambient Conditions. *Chem. - A Eur. J.* **2018**, *24*, 13170–13180. https://doi.org/10.1002/chem.201802240.

(217) Li, J. R.; Tao, Y.; Yu, Q.; Bu, X. H.; Sakamoto, H.; Kitagawa, S. Selective Gas Adsorption and Unique Structural Topology of a Highly Stable Guest-Free Zeolite-Type MOF Material with N-Rich Chiral Open Channels. *Chem. - A Eur. J.* **2008**, *14*, 2771–2776. https://doi.org/10.1002/chem.200701447.

(218) Henke, S.; Schneemann, A.; Wütscher, A.; Fischer, R. A. Directing the Breathing Behavior of Pillared-Layered Metal-Organic Frameworks via a Systematic Library of Functionalized Linkers Bearing Flexible Substituents. *J. Am. Chem. Soc.* **2012**, *134*, 9464–9474. https://doi.org/10.1021/ja302991b.

(219) Mishra, P.; Mekala, S.; Dreisbach, F.; Mandal, B.; Gumma, S. Adsorption of CO 2, CO, CH 4 and N 2 on a Zinc Based Metal Organic Framework. *Sep. Purif. Technol.* **2012**, *94*, 124–130. https://doi.org/10.1016/j.seppur.2011.09.041.

(220) Henke, S.; Fischer, R. a. Gated Channels in a Honeycomb-like Zinc - Dicarboxylate - Bipyridine. *J. Am. Chem. Soc.* **2011**, *133*, 2064–2067.

(221) Henke, S.; Schmid, R.; Grunwaldt, J. D.; Fischer, R. A. Flexibility and Sorption Selectivity in Rigid Metal-Organic Frameworks: The Impact of Ether-Functionalised Linkers. *Chem. - A Eur. J.* **2010**, *16*, 14296–14306. https://doi.org/10.1002/chem.201002341.

(222) Zhang, J.; Wu, H.; Emge, T. J.; Li, J. A Flexible MMOF Exhibiting High Selectivity for CO2 over N 2, CH4 and Other Small Gases. *Chem. Commun.* **2010**, *46*, 9152–9154. https://doi.org/10.1039/c0cc02942d.

(223) Bae, Y. S.; Hauser, B. G.; Farha, O. K.; Hupp, J. T.; Snurr, R. Q. Enhancement of CO2/CH4 Selectivity in Metal-Organic Frameworks Containing Lithium Cations. *Microporous Mesoporous Mater.* **2011**, *141*, 231–235. https://doi.org/10.1016/j.micromeso.2010.10.048.

(224) Vaidhyanathan, R.; Iremonger, S. S.; Dawson, K. W.; Shimizu, G. K. H. An Amine-Functionalized Metal Organic Framework for Preferential CO 2 Adsorption at Low Pressures. *Chem. Commun.* **2009**, 5230–5232. https://doi.org/10.1039/b911481e.

(225) Zhang, Z.; Xiang, S.; Rao, X.; Zheng, Q.; Fronczek, F. R.; Qian, G.; Chen, B. A Rod Packing Microporous Metal-Organic Framework with Open Metal

Sites for Selective Guest Sorption and Sensing of Nitrobenzene. *Chem. Commun.* **2010**, *46*, 7205–7207. https://doi.org/10.1039/c0cc01236j.

(226) Lee, C. H.; Huang, H. Y.; Liu, Y. H.; Luo, T. T.; Lee, G. H.; Peng, S. M.; Jiang, J. C.; Chao, I.; Lu, K. L. Cooperative Effect of Unsheltered Amide Groups on CO2 Adsorption inside Open-Ended Channels of a Zinc(II)-Organic Framework. *Inorg. Chem.* **2013**, *52*, 3962–3968. https://doi.org/10.1021/ic302758g.
